# Silicon protects soybean plants against *Phytophthora sojae* by interfering with effector-receptor expression

**DOI:** 10.1186/s12870-018-1312-7

**Published:** 2018-05-30

**Authors:** Aliyeh Rasoolizadeh, Caroline Labbé, Humira Sonah, Rupesh K. Deshmukh, François Belzile, James G. Menzies, Richard R. Bélanger

**Affiliations:** 1 Département de Phytologie, Faculté des Sciences de l’Agriculture et de l’Alimentation, Université Laval, Québec City, Québec G1V 0A6 Canada; 2 Département de Phytologie and Institue de biologie intégrative et des systèmes, Université Laval, Québec City, Québec Canada; 3 Agriculture and Agri-Food Canada, 101 Route 100, Morden, MB R6M 1Y5 Canada

**Keywords:** Transcriptome, *Glycine max*, Plant receptors, Effectors, Defense genes

## Abstract

**Background:**

Silicon (Si) is known to protect against biotrophic and hemibiotrophic plant pathogens; however, the mechanisms by which it exerts its prophylactic role remain unknown. In an attempt to obtain unique insights into the mode of action of Si, we conducted a full comparative transcriptomic analysis of soybean (*Glycine max*) plants and *Phytophthora sojae*, a hemibiotroph that relies heavily on effectors for its virulence.

**Results:**

Supplying Si to inoculated plants provided a strong protection against *P. sojae* over the course of the experiment (21 day). Our results showed that the response of Si-free (Si^−^) plants to inoculation was characterized early (4 dpi) by a high expression of defense-related genes, including plant receptors, which receded over time as the pathogen progressed into the roots. The infection was synchronized with a high expression of effectors by *P. sojae*, the nature of which changed over time. By contrast, the transcriptomic response of Si-fed (Si^+^) plants was remarkably unaffected by the presence of *P. sojae*, and the expression of effector-coding genes by the pathogen was significantly reduced.

**Conclusion:**

Given that the apoplast is a key site of interaction between effectors and plant defenses and receptors in the soybean-*P. sojae* complex, as well as the site of amorphous-Si accumulation, our results indicate that Si likely interferes with the signaling network between *P. sojae* and the plant, preventing or decreasing the release of effectors reaching plant receptors, thus creating a form of incompatible interaction.

**Electronic supplementary material:**

The online version of this article (10.1186/s12870-018-1312-7) contains supplementary material, which is available to authorized users.

## Background

Soybean (*Glycine max* L. *Merr.*) is economically and agriculturally the most important legume in the world, but its production is compromised by many biotic and abiotic factors. Of primary importance, *Phytophthora sojae* Kaufm. and Gred. [27] can cause annual yield losses as high as $200 million in the USA and $1–2 billion worldwide [31]. *Phytophthora sojae* is a soil-borne plant pathogen belonging to the oomycetes with a restricted host range, including soybean as its primary host. It causes root and stem rot, and pre- and post-emergence damping-off, particularly in flooded soils where the pathogen can disseminate easily because of its flagellated zoospores [45]. It is described as a hemibiotrophic pathogen and it secretes effector proteins (coded by *Avr* genes) to manipulate and invade living host cells during the initial biotrophic stage of infection. In plant-pathogen interactions, effectors are recognized as important virulence factors that are utilized by the pathogen to suppress PAMPs (Pathogen-Associated Molecular Patterns)-Triggered Immunity (PTI) and Effector-Triggered Immunity (ETI) in plants or change host metabolism so that it can easily colonize plant tissues [12, 50]. In response, soybean can carry resistance genes to *P. sojae* (*Rps*), that encode, or are predicted to encode, nucleotide-binding leucine-rich repeat (NB-LRR)-type proteins [19, 26], which are able to recognize the *Avr* effector proteins of *P. sojae* and induce the appropriate defense response [10, 18]. The result of this interaction between *Rps* genes and *Avr* genes will often determine compatible or incompatible interactions.

Numerous studies have highlighted the prophylactic effects of silicon (Si) fertilization [3, 16, 20] in the search for additional methods to prevent losses in the case of compatible interactions. Interestingly, Si appears to be particularly efficient against biotrophic and hemibiotrophic fungal/oomycete pathogens [5, 46]. In the case of soybean, Arsenault-Labrecque et al. [1] have shown that a Si treatment was effective against soybean rust caused by the biotrophic fungus *Phakopsora pachyrizi*. In addition, Deshmukh et al. [9] have identified Si transporters in soybean, thus confirming that the species is receptive to Si and can absorb the element.

The mechanisms inherent to the prophylactic properties of Si have puzzled scientists for many years. Originally, it was suggested that Si deposition along the cell walls created a physical barrier that halted fungal penetration into the plant [47]. However, additional studies have linked the presence of Si with diverse plant-defense reactions, thus suggesting that Si may play a role in the induction of acquired resistance [6, 7, 16, 17, 53]. In a recent study using *Arabidopsis thaliana* mutants deficient in salicylic acid (SA) synthesis, Vivancos et al. [46] showed that Si protected both mutant and wild-type plants against powdery mildew (*Erisyphe cichoracearum*). This led the authors to suggest that the deposition of Si as amorphous gel in the apoplast may prevent fungal effectors from reaching their targets, thereby altering the development of the pathogen. This hypothesis becomes particularly relevant in the context of the *P. sojae*-soybean interaction in light of recent results. Indeed, Ma et al. [34] recently showed that *P. sojae* employed an apoplastic decoy strategy with effectors to attack soybean. Xin et al. [52] further proposed that an aqueous apoplast was required for pathogenicity rather than immunosuppression, a condition that can be altered by silicon’s presence. Finally, Wang et al. [50], on the basis of recent results with *P. sojae*, described the apoplastic region as a major battle ground between pathogen effectors and the host apoplastic surveillance system.

Since *P. sojae* is a hemibiotrophic pathogen that relies heavily on effectors for its virulence, the *P. sojae*-soybean pathosystem was deemed well-suited to validate and investigate the hypothesis that Si deposition altered the release of virulence factors by *P. sojae*. In this context, two main objectives were defined: 1) to assess resistance of soybean plants to *P. sojae* when fertilized with Si, and 2) to analyze the expression of salient genes involved in the virulence of *P. sojae* and the defense mechanisms of soybean in order to assess if a differential response could be linked to the prophylactic role of Si.

## Results

### Phenotypic responses

**Fig. 1:**
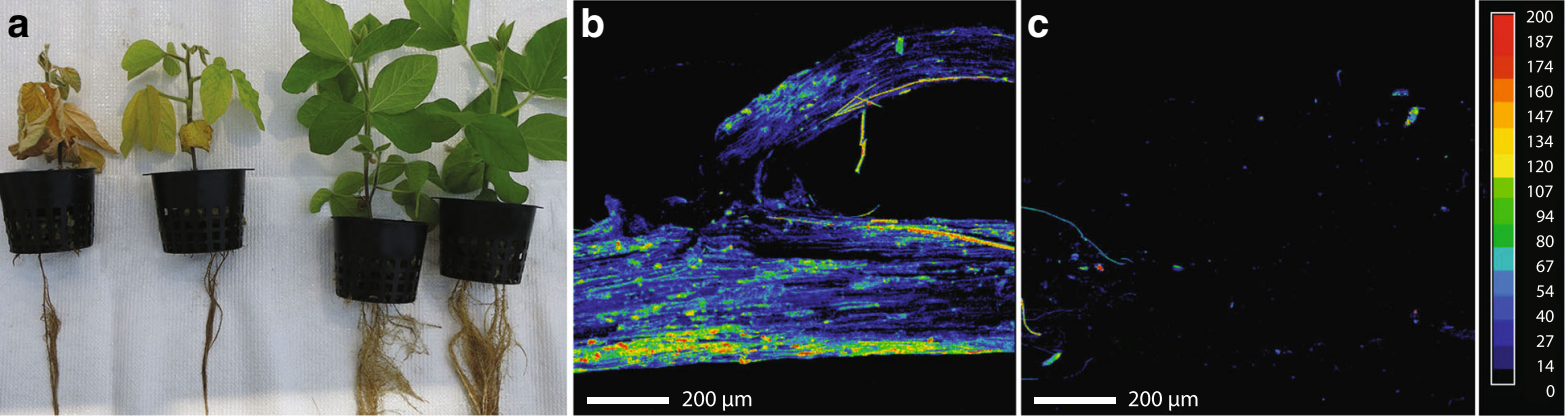
Effect of silicon (Si) amendments on soybean plants 21 days after inoculation with *Phytophthora sojae*. **a** Plants in the Si^+^ treatment were clearly healthier than non-treated plants with more developed roots, stems and leaves. Comparative X-ray superimposed scanning electron micrographs of soybean root tips in plants treated (**b**) or not (**c**) with Si. At least, five plants per treatments were observed. A color scale of Si deposition was used, with blue indicating low Si and red high Si deposition. Black areas indicated no Si deposition

Soybean plants were inoculated with zoospores of *P. sojae* in a recirculating hydroponic system fed with nutrient solution with and without 1.7 mM Si to compare the phenotypic differences linked to Si. First symptoms of root browning appeared as early as 4 days post inoculation (dpi). Stunting and leaf discoloration followed within a few days, and first cases of mortality were recorded at 15 dpi in the Si^−^ treatment. The differences between Si^−^ and Si^+^ treatments increased with time, and by 21 dpi, plants in the Si^+^ treatment were clearly healthier than non-treated plants (Fig. 1a). In terms of dry weight, for non-inoculated plants there was no significant difference between Si^−^ (8.4 ± 0.5) and Si^+^ plants (8.7 ± 0.4) plants. However, inoculation with *P. sojae* significantly reduced plant dry weight, but the prophylactic effect of Si was quite apparent as plants were significantly heavier in the Si^+^ (5.0 g ± 1.9) compared with the Si^−^ (2.0 g ± 1.0) treatment. X-ray microanalysis mapping of soybean confirmed the accumulation of Si throughout the roots in Si^+^ plants (Fig. 1b), while, in the absence of Si amendment, no clear evidence of Si deposition was observed (Fig. 1c).

### Dual RNA-seq analysis of the *P. sojae*-soybean interaction in the presence of Si

A complete comparative transcriptomic analysis of soybean roots and *P. sojae* was carried out at 0, 4, 7 and 14 dpi to obtain a comprehensive gene-expression profile for both soybean and *P. sojae* in response to Si application.

### Soybean root transcriptome

**Fig. 2:**
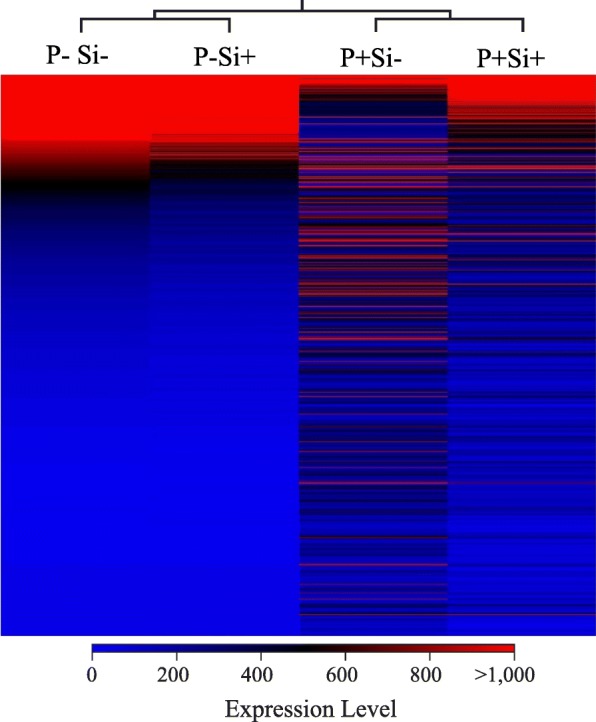
Heat map of differentially expressed genes in *Phytophthora sojae* infecting soybean roots. In total, 3294 genes were differentially expressed as a result of *P. sojae* infection at 4 dpi (P^+^Si^−^). Heat map shows gene expression pattern in soybean roots inoculated (P^+^) or not (P^−^) with *P. sojae* and treated (Si^+^) or not (Si^−^) with silicon. Each gene corresponds to a colored line indicating the normalized mean (*n* = 5) of the differentially expressed transcripts (Fold-change ≥4, FDR *p*-value ≤0.01)

Mapping of the processed reads from roots to the soybean genome showed a very high percentage of mapped reads for non-inoculated samples (control) treated or not with Si. For control plants, 81 and 90% of reads mapped on soybean in Si^−^ and Si^+^ treatments, respectively. In inoculated plants at 4 dpi, 61 and 76% of reads mapped to soybean in Si^−^ and Si^+^, respectively (Additional file 1: Table S1). Interestingly, the number of differentially expressed genes (DEGs) between control plants treated or not with Si was limited to 50 out of the potential 56,045 genes analyzed, and all were downregulated in the Si^+^ treatment (Fold-change ≥4, FDR *p*-value ≤0.01). On the other hand, plants responded to inoculation of *P. sojae* (Si^−^P^+^ vs. Si^−^P^−^) with a differential expression of 3294 genes (Additional file 2: Table S2). Most of genes that were differentially expressed as a result of the infection (Si^−^P^+^ vs Si^−^P^−^) reverted to a pattern of expression closer to control plants in the Si^+^ treatment (Si^+^P^+^ vs Si^−^P^+)^ as illustrated on the heat map (Fig. 2).

Functional categorization of the DEGs in *P. sojae*-infected plants showed that these genes belonged mainly to the following categories: defense-related genes, secondary metabolism, hormone metabolism, primary metabolism and no-ontology for which no function was annotated.

#### Defense-related genes

Most known pattern recognition receptors (PRRs) that can activate PTI in plants fall into one of two receptor classes: transmembrane receptor kinases and receptor-like kinases (RLK; [10, 29, 32]). In our study, 46 DEGs belonged to the receptor kinase family and 24 RLK showed higher expression at 4 dpi in the Si^−^ treatment (Additional file 3: Figure S1 a, b). After PRR activation, the downstream signaling pathway transfers signals from extracellular receptors to cellular responses by mitogen-activated protein kinases (MAPKs) and calcium (Ca^2+^). MAPKs are ubiquitous signal-transduction components, which have been implicated in both PTI and ETI. Our results showed that out of nine differentially-expressed MAPKs, five had a higher expression at 4 dpi in the Si^−^ treatment (Additional file 3: Figure S1c). Similarly, 33 Ca^2+^-dependent protein kinases (CDPKs) were highly expressed at 4 dpi (Additional file 3: Figure S1d, Additional file 4: Table S3).

**Fig. 3:**
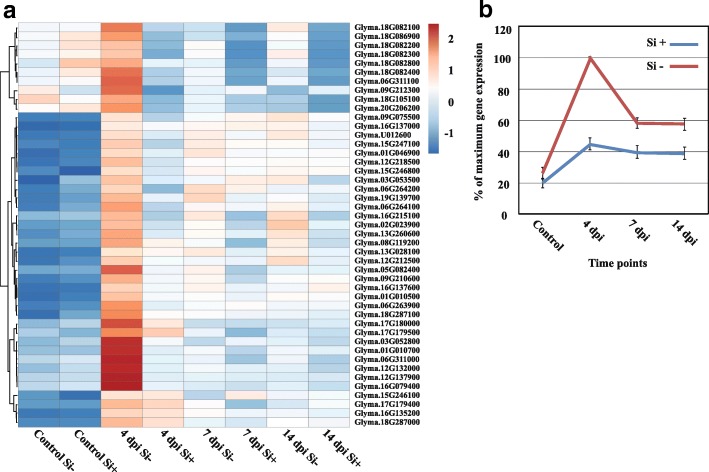
Expression profile of *NB-LRRs* genes. Heat map (**a**) and gene expression (**b**) show a higher expression of 45 receptor (*NB-LRR*) genes in *Phytophthora sojae*-inoculated soybean plants at 4 dpi under Si^−^ compared to Si^+^ treatment. **b** Graph shows the average relative (%) expression at each timepoint based on the highest level of expression for each gene as a measure to showcase the trend in expression. Bars represent standard error from the mean (*n* = 5)

NB-LRR proteins. Out of 80 differentially-expressed *NB-LRR* genes over the experimental period (Fold-change ≥4, FDR *p*-value ≤0.01), 45 showed their highest expression at 4 dpi in Si^−^ plants. Heat map results clearly showed a pattern of expression where there was no expression of *NB-LRR* genes in non-inoculated plants (control) regardless of Si treatment, followed by a sharp increase at 4 dpi in Si^−^ plants. While the expression was reduced at 7 and 14 dpi, it remained significantly higher in Si^−^ plants (Fig. 3, Additional file 5: Table S4).

**Fig. 4:**
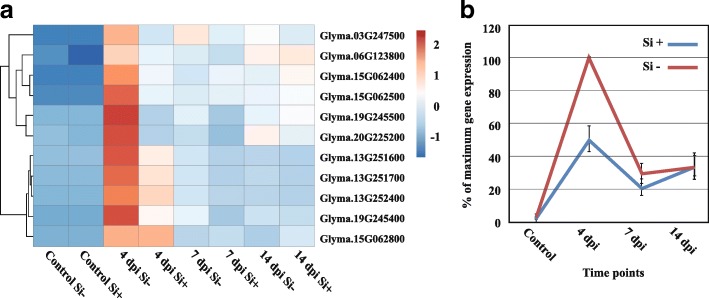
Expression profile of *PR* genes. Heat map (**a**) and gene expression (**b**) show a higher expression of 11 pathogenesis-related (*PR*) genes in *Phytophthora sojae*-inoculated soybean plants at 4 dpi under Si^−^ compared to Si^+^ treatment. **b** Graph shows the average relative (%) expression at each timepoint based on the highest level of expression for each gene as a measure to showcase the trend in expression dynamics. Bars represent standard error from the mean (*n* = 5)

Pathogenesis-related proteins (PRs). Based on cluster analysis, 11 *PR* genes were found to be differentially expressed in at least one timepoint (Fold-change ≥4, FDR *p*-value ≤0.01). Incidentally, heat-map results clearly showed that 4 dpi was the critical timepoint differentiating Si^−^ and Si^+^ plants in terms of *PR*-gene expression (Fig. 4, Additional file 6: Table S5). The expression receded over time (7 and 14 dpi) to similar levels between the treatments.

Transcription Factors Associated with Defense Expression. Out of the 67 differentially-expressed WRKY transcription factors in at least one timepoint, most showed a higher expression in Si^−^ treatment, with 20 genes showing a higher expression at 4 dpi in the Si^−^ treatment (Additional file 7: Figure S2, Additional file 8: Table S6).

Miscellaneous defense responses. Other genes linked to defense responses showed a similar pattern of expression where they were quickly upregulated at 4 dpi in Si^−^ plants before receding to levels similar to those observed in Si^+^ plants. For instance, of the 16 differentially-expressed plant protease inhibitors in at least one timepoint, 13 showed higher expression in the Si^−^ treatment at 4 dpi. Similarly, out of the seven differentially-expressed polyphenol oxidases in at least one timepoint, five showed higher expression at 4 dpi in the Si^−^ treatment (Additional file 9: Figure S3, Additional file 10: Table S7).

#### Secondary metabolism

We observed 31 genes involved in flavonoid metabolism with a higher expression at 4 dpi in the Si^−^ treatment. We also observed nine genes involved in isoflavone metabolism and 15 genes involved in isoprenoid metabolism, all with higher expression at 4 dpi in the Si^−^ treatment (Additional file 11: Figure S4, Additional file 12: Table S8).

#### Hormone metabolism

**Fig. 5:**
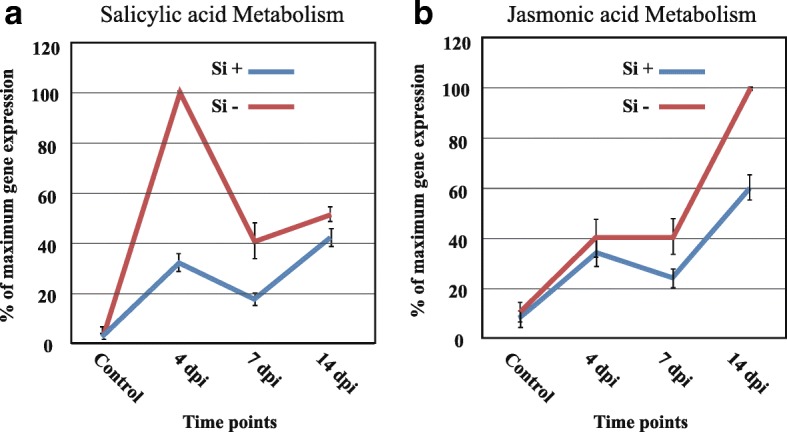
Expression profile of hormone-related genes. Gene expression shows a higher expression of five genes involved in SA metabolism (**a**), 13 genes involved in JA metabolism (**b**) in *Phytophthora sojae*-inoculated soybean plants at 4 dpi under Si^−^ compared to Si^+^ treatment. Graph shows the average relative (%) expression at each timepoint based on the highest level of expression for each gene as a measure to showcase the trend in expression dynamics. Bars represent standard error from the mean (*n* = 5)

In our system, five genes involved in SA metabolism had higher expression at 4 dpi in the Si^−^ treatment, in synchrony with the biotrophic phase of *P. sojae,* and 13 genes involved in JA metabolism were differentially expressed with higher expression at 14 dpi in the same treatment (Fig. 5, Additional file 13: Table S9), a period more in-line with the necrotrophic development of the pathogen.

#### Primary metabolism

Based on functional categorization, primary metabolism included the most categories that contained DEGs. Out of 580 DEGs involved in primary metabolism as a result of *P. sojae* infection in the Si^−^ treatment, 70% were upregulated and 30% were downregulated. In the Si^+^ treatment, the number of DEGs was reduced to 420 genes with a similar proportion of up- and down-regulation. Upregulated transcripts were associated with processes involved in energy production, such as carbohydrate metabolism, TCA cycle, gluconeogenesis, mitochondrial electron transport, ATP biosynthesis and amino acids biosynthesis, as well as biosynthesis of lipid metabolism (Additional file 14: Figure S5).

#### *Phytophthora sojae* transcriptome

**Fig. 6:**
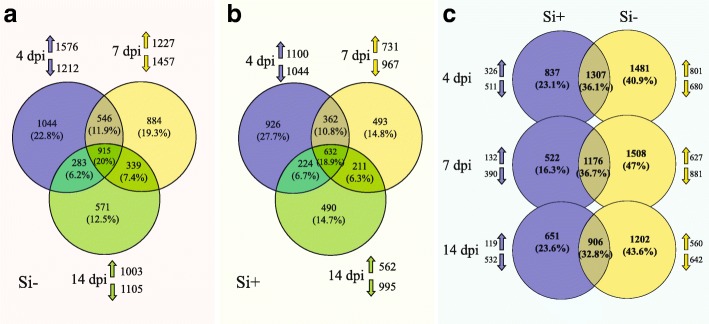
Venn diagram. Graphs show the number of differentially expressed genes (DEGs) in *Phytophthora sojae* infecting soybean in comparison with *P. sojae* in axenic culture. Number of DEGs (Fold-change ≥4, FDR *p*-value ≤0.01) observed in Si^−^ plants (**a**), Si^+^ plants (**b**) at 4 (blue), 7 (yellow) and 14 (green) dpi. and (**c**) comparative number of DEGs in *P. sojae* between Si + (blue) and Si- (yellow) plants at 4, 7, 14 dpi. The number of unique DEGs was consistently higher in Si- plants than in Si + plants and the highest at 4 dpi. The up and down arrows indicate the number of up- and down- regulated genes, respectively, for each timepoint

More than 90% of the processed reads from the five biological replications of *P. sojae* cultured in vitro mapped to the *P. sojae* genome (Additional file 15: Table S10). To determine which genes were differentially expressed throughout the interaction with soybean, we compared gene expression *in planta* with axenic samples. The number of DEGs in *P. sojae* was higher under Si^−^ compared to Si^+^ conditions (Fig. 6). The highest number of DEGs per treatment (Si^−^ or Si^+^) was recorded at 4 dpi, and this number kept receding over time.

#### Annotation

When looking at the top 100 upregulated DEGs in *P. sojae* over all timepoint and treatments, most were linked to hypothetical proteins for which functional annotation was not available (Additional file 16: Table S11). A notable exception was *Avh1b-81*, which ranked among the highest expressed genes at 4 and 7 dpi in the Si^−^ treatment.

#### Expression of effectors in *P. sojae* during Si^−^ and Si^+^ treatments

**Fig. 7:**
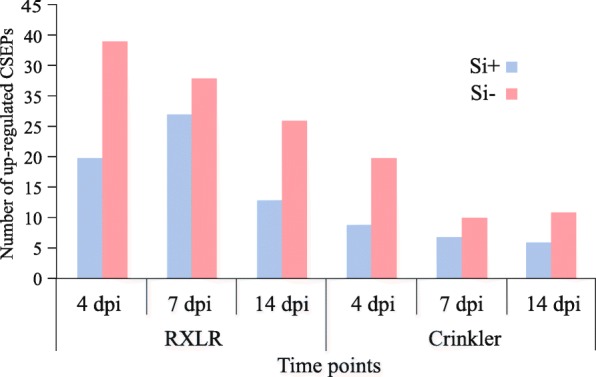
Number of upregulated effector genes in *Phytophthora sojae* over time. Cluster comparison of *P. sojae* effectors in association with silicon-treated (Si^+^) or untreated (Si^−^) soybean plants shows the larger number of upregulated Crickler and RxLR effector genes in Si^−^ plants compared to Si ^+^ plants, particularly at 4 dpi

In oomycetes, many effectors are characterized by an RxLR motif [11]. Of the 348 RxLR effector genes identified in *P. sojae,* 104 were found to be differentially expressed in at least one timepoint (Fold-change ≥4, FDR *p*-value ≤0.01; Fig. 7). Time-series analysis showed a higher number of upregulated RxLR effectors at 4 dpi in Si^−^ plants, in line with observations of first symptoms in soybean plants. On the other hand, while the number was always higher in Si- plants, there were more RxLR effectors expressed in Si + plants at 7 dpi than at 4 dpi.

Crinkler (CRN) effectors are another group of important secreted effectors by *P. sojae*. Of the 226 CRN effector genes identified in *P. sojae*, 21 were found to be differentially expressed in at least one timepoint (Fold-change ≥4, FDR *p*-value ≤0.01; Fig. 7). Cluster comparison showed a higher number of upregulated CRN effectors in Si^−^ plants compared to Si^+^ plants and particularly at 4 dpi. Interestingly, the recently reported apoplastic effector *PsXEG1* and its decoy (*PsXLP1*; [34]) had no expression under our experimental conditions.

**Fig. 8:**
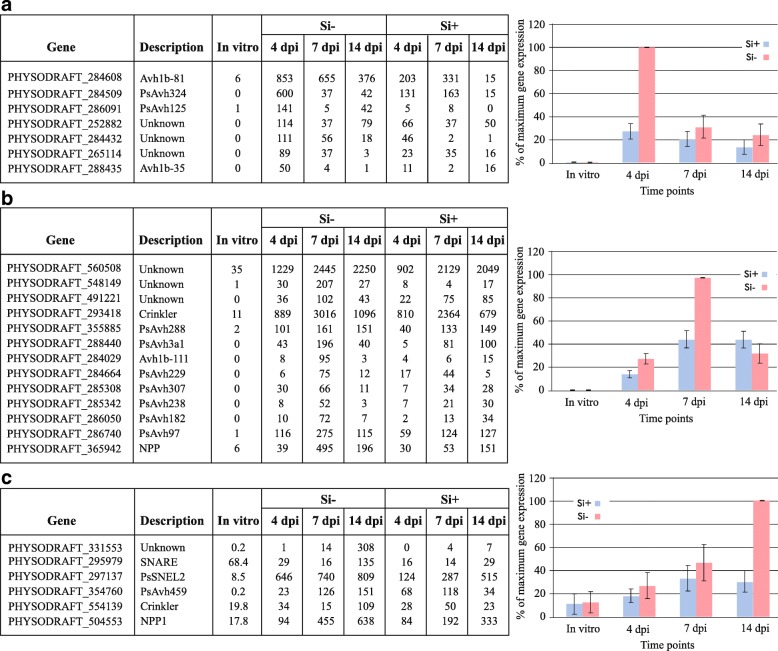
Comparison of differentially expressed *Phytophthora sojae* CSEPs over time. Tables include normalized mean of the most differentially expressed CSEPs genes compared to axenic cultures specific to *P. sojae* at (**a**) 4 dpi, (**b**) 7 dpi and (**c**) 14 dpi (Fold-change ≥ 4, FDR *p*-value ≤ 0.01). Hierarchical cluster analysis at (**a**) 4 dpi, (**b**) 7 dpi and (**c**) 14 dpi shows a systematically higher level of expression in Si-soybean plants than in Si+ plants. Each graph shows the average relative (%) cluster expression at each timepoint based on the highest level of expression for each gene as a measure to showcase the trend in expression dynamics. Bars represent standard error from the mean (*n* = 5)

Hierarchical cluster analysis was performed on *P. sojae* candidate secreted effector proteins (CSEPs) in order to identify genes with similar expression profiles at different timepoints. Our results showed that some CSEPs were clearly more expressed at 4 dpi and particularly in the Si^−^ treatment (Fig. 8a). In the same manner, other CSEPs were preferentially expressed at 7 dpi (Fig. 8b), while others were at the later stage of infection (Fig. 8c), and the higher expression was always associated with the Si^−^ treatment. Notably, these CSEPs showed no expression in axenic culture. Interestingly, only CSEPs with an RxLR motif were highly expressed at 4 dpi, while CRN effectors and members of other groups, such as necrosis-inducing proteins, were highly expressed at 7 and/or 14 dpi (Fig. 8).

## Discussion

A full comparative transcriptomic analysis of the effect of Si on *P. sojae*-infected soybean plants in this study suggests that Si may protect plants by interfering with the dialogue between pathogen effectors and plant defenses receptors, thus preventing a compatible interaction. To date, the protective effect of Si against a various range of plant pathogens has been well documented [3, 16, 46], but many questions regarding how it exacts its role on plant defenses remain unanswered. The mechanical barrier impeding fungal penetration was the first explanation of the protective role of Si [47], but this hypothesis has been slowly abandoned, namely based on results showing no sufficient increase in leaf toughness to retard fungal penetration following Si application [7, 54]. Cherif et al. [6, 8] were the first to report the induction of defense responses such as lignin, phenolic compounds and phytoalexins in association with root application of Si on cucumber plants infected by *Pythium ultimum*, an observation that has since been reproduced in other host-pathogen interactions [16, 17, 20, 40]. However, Vivancos et al. [46] recently reported that mutant Arabidopsis plants unable to mount defense reactions through the SA pathway were still protected against powdery mildew (*Erisyphe cichoracearum*) when fed with Si. This suggested that, unlike the mechanism proposed by Fawe et al. [17], the role of Si was not solely linked to the activation of defense responses, as supported by results from this study.

Our data have clearly established that the Si treatment offered a good protection of soybean plants against *P. sojae*, confirming a previous report by Guérin et al. [21]. From a practical point of view, biotrophic and hemibiotrophic (such as *P. sojae*) pathogens dominate the list of pathogens being controlled by Si, and the effect appears more durable and less transient than what is reported with necrotrophic pathogens. Given that the former pathogens are usually more species-specific in their host range, this led Vivancos et al. [46] to suggest that Si may somehow interfere with host recognition.

The comparative transcriptomic analysis of the infection process of *P. sojae* on soybean, as influenced by Si, has revealed an intricate pattern of gene expression by both *P. sojae* and the plant along with unprecedented insights into the mechanisms by which Si can protect plants. From the onset, it was interesting to observe that the effect of Si amendment on control plants was negligible, where only 50 out of the ca. 56,000 soybean transcripts were expressed differentially. Given that most were downregulated with no clear pathways being influenced, this reinforces the notion that the beneficial effects of Si are strictly protective or stress-related in nature. These results corroborate previous studies on Arabidopsis-*Erysiphe cichoracearum* [15], wheat-*Blumeria graminis f. sp. tritici* [5] and rice [51], where the effects of Si were nil or minimal in absence of a stress. On the other hand, they contradict other studies claiming that Si feeding alone can improve plant growth [24, 33, 35, 48], even though this concept is being challenged by many studies, including the present one.

Soybean plants infected with *P. sojae* displayed an active transcriptome where over 3000 genes were differentially expressed compared to control plants, especially in the primary metabolism and stress-defense categories. These observations are in line with a recent study on soybean- *Fusarium oxysporum* by Lanubile et al. [29], where they found the largest portion of DEGs assigned to these two categories. While the role of primary metabolism as an energy provider is undeniable, its role on regulation of defense responses in plants has been well documented in many studies [41]. High expression of genes involved in primary metabolism can be associated with hemibiotrophic pathogens hijacking plant metabolism for their own benefits, thus requiring a greater expense of energy from the plant. The up-regulation of primary metabolism is consistent with previous results on wheat-*Blumeria graminis f. sp. tritici* in Si^−^ treatment [5], where the authors associated this up-regulation with the presence of the biotrophic pathogen, and in the case of the soybean-*Fusarium oxysporum* interaction [29], the high activation of primary metabolism was associated with the increased demand for energy to induce defense-related genes.

The high expression of defense-related genes in infected plants gives evidence of attempts by soybean plants to fend off *P. sojae*, especially during the early stage of infection (4 dpi), while the expression was reduced at 7 and 14 dpi as the pathogen progressed within the roots. The same result has been observed in response to a pathogenic isolate of *Fusarium oxysporum*, where soybean accumulated more defense-response transcripts in the first days following infection while these responses eventually receded [29]. This chain of events has been reported in previous studies including soybean-*P. sojae* interaction [36], Arabidopsis-*E. cichoracearum* [15], wheat-*Blumeria graminis f. sp. tritici* [5] and soybean-*Fusarium virguliforme* [38], in which it was suggested that the first reaction of plants to pathogens was the activation of an array of defense-related genes, even in the case of compatible interactions. In the same manner, at 4 dpi in Si^−^ plants, the higher activation of NB-LRR receptors, which are known as markers of activation of effector-triggered immune (ETI) responses, led nevertheless to a compatible interaction as opposed to Si^+^ plants. This shows that expression of defense-related genes does not necessarily translate into a resistance response. For instance, the higher activation of NB-LRRs has been also reported in the compatible soybean-*Fusarium virguliforme* interaction [38]. The pattern of expression for other pathways related to defense reactions was similar where higher expression was consistently observed at 4 dpi in Si^−^ plants. In line with this pattern, our results demonstrated the transcriptional induction of multiple genes involved in secondary metabolism and signaling. The same observations were reported in soybean with the pathogens *P. sojae* [36], *F. virguliforme* [38], and *F. oxysporum* [29]. Our work further showed that infection by *P. sojae* triggered SA metabolism during the early stages of infection while JA metabolism was more activated during the latter stages of infection, especially in Si^−^ plants. This pattern of gene expression has been reported previously in interactions between plants and hemibiotrophic pathogens such as canola and *Leptosphaeria maculans*, and soybean and *P. sojae* [21, 36, 42].

Treatment of soybean plants with 1.7 mM Si provided a strong protection against *P. sojae,* a result supported by the phenotypes and the heat map, whereby the overall transcriptomic response of the infected plants treated with Si was somewhat similar to that of control plants. These results corroborate those of Chain et al. [5], who showed that Si-treated plants were basically unresponsive to the presence of *E. cichoracearum*.

To better understand this phenomenon, the transcriptomic response of *P. sojae* on both control and Si^+^ plants was followed and compared over time. As reported in many studies, hemibiotrophic pathogens will secrete CSEPs into the apoplastic region of plant cells, that can either act directly in the apoplast or be translocated into the cell, to neutralize plant defense reactions during the biotrophic stage and induce necrosis during the necrotrophic phase [49]. Cluster comparison of differentially-expressed *P. sojae* CSEPs at different timepoints showed a systematically higher number in Si^−^ plants compared to Si^+^ plants associated with a higher expression of specific CSEPs at 4 dpi in the Si^−^ treatment, a result that corroborates the higher expression of NB-LRRs at the same timepoint. For instance, Avh324, previously reported as an effector involved in the biotrophic phase of *P. sojae* [49], was one of the most highly expressed CSEPs at 4 dpi in our study. Interestingly, our results showed both a fewer number of RxLR effectors upregulated in Si + plants, and a delayed pattern from 4 to 7 days in Si + plants compared to Si- plants. These results are perfectly congruent with a multitude of previous reports showing that Si will delay the onset of disease and reduce its magnitude [6, 7, 15, 20, 22]. Other effectors categorized as necrosis-inducing effectors, such as Avh238 [49], were found to be highly expressed in the later stages of infection in Si^−^ plants. The relative down-regulation of CSEPs in incompatible interactions has been reported in Arabidopsis*-Blumeria graminis f. sp. hordei* [25]. These results are well in agreement with the phenotypic differences observed between Si^−^ and Si^+^ plants.

Our results clearly demonstrate that feeding soybean plants with Si 7 days before inoculation with *P. sojae* resulted in some protection against infection and a systematic deposition of Si in soybean roots (see Fig. 1). It has been well demonstrated that Si will deposit in plants in the form of amorphous silicon primarily in the apoplastic region of plant cells [2, 55]. As mentioned previously, the apoplast is a privileged site of initial release or activity for secreted CSEPs [37]. The apoplast plays a crucial role in plant-pathogen interactions as it will dictate the establishment or suppression of a pathogen based on the interaction between pathogen effectors and plant defenses and receptors [4, 50, 52]. Furthermore, filamentous pathogens/oomycetes will often release effectors initially in the apoplast before they get translocated inside the cell where they can interact with other receptors such as NLRs [37]. Along these lines, recent reports have emphasized the particular importance of the apoplastic region for the development of *P. sojae*. Most notably, Ma et al. [34] showed that *P. sojae* employed an apoplastic decoy strategy where *Avr* gene products elude recognition by receptors thus inhibiting defense responses. Furthermore, Xin et al. [52] proposed that water availability in the apoplast played a key role in the ensuing infection by *P. sojae*. Taken together, our results showed that Si feeding led to a form of incompatible interaction between soybean and *P. sojae* and suggest that the presence of Si in the apoplast is possibly linked to its prophylactic properties. As a first evidence, polymerization of Si in the apoplast is known to reduce the abundance of free water [19], which would change the apoplastic environment and make it less conducive for *P. sojae* development. Secondly, the presence of amorphous Si along the plasma membrane could a) restrict the required signals from the plant toward the pathogen which act to induce cell differentiation and express essential pathogenicity genes, b) interfere with the signaling flow between *P. sojae* and the plant, preventing or decreasing, for instance, the release of effectors reaching plant receptors, c) interfere with the translocation of effectors into the plant cell and d) confine the transition of nutrients from host toward hemibiotrophic pathogen, leading to a form of non-host resistance [28, 44]. Incidentally, Vivancos et al. [46]. showed that even SA-deficient Arabidopsis mutants were resistant to powdery mildew when fed with Si and suggested that inhibition of effector release explained that result.

## Conclusion

Our work presents novel insights into the mechanistic role by which Si deposition influences the outcome of host-pathogen interactions. More specifically, results showed that a Si treatment conferred a good protection of soybean plants against *P. sojae*. The transcriptome analysis revealed that Si-treated plants had a surprisingly lower defense response than Si-deprived plants, and that *P. sojae* had a much lower diversity and intensity of effector transcripts on Si^+^ plants. These results support the hypothesis that Si interferes with the signaling process between a plant and a biotrophic/hemibiotrophic pathogen to elicit an incompatible interaction.

## Methods

To evaluate the phenotypic responses of soybean plants to a Si treatment, we used the methodology developed by Guérin et al. [21]. Plants were grown in hydroponic systems with four different treatments: soybean plants inoculated with *P. sojae* and grown with and without Si (pH 7.0) in the form of potassium silicate, and control plants (without *P. sojae*) grown with and without Si. In all experiments, Si concentration was adjusted to 1.7 mM as it represents the highest possible concentration of silicic acid in solution and it is the standard procedure used to test the prophylactic role of Si [46]. Each treatment included two separate troughs fed with a nutrient solution. For each treatment and each trough, five plants were used for a total of 10 plants per treatment.

### Plant growth conditions

Soybean (*Glycine max* cv. Hikmok sorip obtained from GRIN (PI 372415)) was selected based on the consistent phenotypic characteristics observed over several generations and its ability to absorb Si and its lack of *Rps* genes [1, 22]. The genotype used in present study was developed at University Laval. For the RNA-seq and disease assay, seeds collected from plants raised at Laval University greenhouse were used after ensuring genetic purity. Seeds were immersed in 5% sodium hypochlorite for 1 min, followed by three subsequent washes with distilled water. Then, seeds were planted in Oasis cubes for 4–5 days in the greenhouse. After emergence of the hypocotyl and the roots, every Oasis cube containing one plant was transferred to the hydroponic system. Plants received water for 2 days, and on the third day solutions containing macro- and micronutrients were added to the trough (see below), Fe Na EDTA and Si in the form of potassium silicate (Kasil #6, 23.6% SiO_2_; National Silicates, Quebec, QC, Canada). After 7 days, zoospores of *P. sojae* were added to the tanks. The macronutrients (KNO_3_, KCl, CaCl_2_, K_2_HPO_4_, MgSO_4_·7H_2_O, MgCl_2_ 6H_2_O) were prepared as a 30X solution; micronutrients (H_3_BO_3_, MnSO_4_·H_2_O, CuSO_4_·5H_2_O, ZnSO_4_·7H_2_O, NaMoO_4_·2H_2_O, Co(NO_3_)_2_·6H_2_O) as a 5,000X solution; and FeNa-EDTA (13.2%) was separately prepared as a 3000X solution. A 50-L solution containing 2 l of macronutrients, 12 ml of micronutrients and 19.8 ml of Fe-EDTA, and adjusted to pH 6.5, was added to 60-L trough.

### *Phytophthora sojae* inoculation

For *P. sojae* inoculation, we followed the procedure recently described by Lebreton et al. [30]. The isolate of *P. sojae* was obtained from the bank collected and maintained by A. Xue at AAFC, Ottawa. Pieces from the “long-term inoculum” of *P. sojae* isolate ont-42-1 (pathotype 1a, 1c, 1d, 1 k and 7) were transferred into Petri dishes containing V8 agar, sealed with parafilm and incubated at 28 °C. After 6 days, 60 mycelial plugs (9 mm diameter) were immersed in a Petri dish (150 × 150 mm) in 60 ml of sterile tap water added to 15 ml of sterile Agromix soil extract. Five plates were sealed with Parafilm and gently shaken on an orbital shaker at room temperature for 18–24 h. Zoospores were observed with a microscope, and swimming and encysted zoospores were counted. A 1-μl drop was placed on a glass slide and observed under a 100X microscopic field. Ten observations were used to determine the zoospore concentration in suspension, and cultures ranging between 10^3^ to 10^4^ zoospores/ml were used for inoculation. Five plates of mycelial plugs provided ca. 350 ml of zoospore suspension that were collected in a 500-ml bottle and added directly to the nutrient solution in 60-l tanks. The spore suspension or an equal volume of water for control plants was added to the tanks 7 days after transfer of seedlings to the hydroponic system.

The plants were monitored daily to record symptom development. After 21 days, plants were harvested and dried at 65 °C for 24 h to determine their dry weight.

For axenic cultures of *P. sojae*, cellophane papers were placed on the top of V8 agar containing Petri dishes and the pieces from the same “long-term inoculum” of *P. sojae* isolate ont-42-1 were transferred on the paper in V8 agar culture, sealed with parafilm and incubated at 28 °C. After 6 days, mycelium of *P. sojae* was removed from the paper, lyophilized and ground for RNA extraction.

### Microscopic and X-ray analyses

X-ray microanalysis mapping was used to locate Si deposition in soybean roots fed with and without Si. At least five root samples per treatment were prepared as described by Guével et al. [23]. Briefly, roots were lyophilized and coated with gold and palladium to provide conductivity to the samples. Samples were analyzed using a CAMECA SX-100 Universal EPMA microscope (Cameca instruments Inc., www.cameca.com) operating at a voltage of 15 kV and a current of 20 nA.

### RNA extraction, library construction and sequencing

Five separate soybean plants (biological replications) per treatment were grown in the hydroponic system. For each treatment, 3-cm samples of top roots were harvested from each of the treated plants and this process was repeated at each timepoint. In preliminary experiments, results showed that first symptoms [30] and first *P. sojae* reads were consistently detectable after 4 days in the hydroponic system. Our sampling protocol was adjusted accordingly. Root tissues were placed in the liquid nitrogen until transfer to the lab and stored at − 80 C. Total RNA was extracted from five biological replicates of root samples that were collected at four timepoints. [0 (pre- inoculation), 4, 7 and 14 days post-inoculation] and five biological replicates of *P. sojae* cultivated in axenic culture, using TRIzol and RNeasy mini kit (Qiagen) including DNAse treatment as per the manufacturer’s instructions. RNA quality and concentration were checked by agarose gel electrophoresis, spectrophotometry (Nanodrop ND-1000) and ultimately, by an Agilent 2100 Bioanalyzer™ (Agilent Technologies).

RNA-seq libraries were generated using the Lexogen® RNA-Seq Sample Preparation kit according to the manufacturer’s protocol (Illumina Inc.). The Lexogen® is designed to generate Illumina compatible libraries from polyadenylated RNA and generate only one fragment per transcript. The efficiency of the cDNA library was measured by an Agilent 2100 Bioanalyzer™ and sequencing was carried out on an Illumina HiSeq™ 2000 platform.

### RNA-Seq data analysis

#### Raw reads processing and high-quality reads alignment to the reference genomes

Poly-A, adaptor sequence contaminants and low-quality bases (Q < 15) were trimmed from Illumina reads in FASTQ format using the RNA-seq analysis tool of CLC Genomics Workbench V.9.5.4 (CLC Bio, Aarhus, Denmark) before further processing. All cleaned reads >40 bp in length were aligned to the PLANAT ribosomal RNA sequence to eliminate unexpected ribosomal RNA from reads; the remaining unmapped reads were then aligned to the soybean reference genome (*Glycine max* Wm82.a2. v1) and then against the *P. sojae* reference genomes (*Phytophthora sojae* V3). The criteria used to map the unique sequence reads included: the mismatch cost of 2, insert cost of 3, deletion cost of 3, minimum length fraction of 0.9 and minimum similarity fraction of 0.8.

#### Gene expression, differential gene expression analyses and clustering

The normalized expression values were taken to estimate the expression levels. The differentially expressed genes (DEGs) were identified using the EdgeR algorithm implemented in CLC Genomics Workbench that utilizes the Exact Test developed by Robinson and Smyth [39]. The *p*-value threshold was determined by the false discovery rate (FDR) to account for multiple tests of significance. To judge the significance of the gene expression, change during plant-pathogen interactions, a FDR threshold ≤0.01 was adopted. The absolute of log 2 (Fold change) > 4 relative to control conditions was used to select up- and/or downregulated genes. To identify general trends during the interaction, a hierarchical clustering of features was also generated for each condition (Si^+^/Si^−^) using normalized expression values. Given that RNA-seq analysis has been conducted on more than three replicates for each treatment, qPCR validation was deemed unnecessary [14].

#### Functional annotation and gene ontology

The web-based Blast2GO and AgriGO tools [13] were used to obtain Gene Ontology (GO) annotations and to perform singular enrichment analyses (FDR *p*-value ≤0.05) of genes of soybean and *P. sojae* differentially expressed during the interaction. Significant enrichment testing was performed for GO categories and Mercator bins with Mapman [43] to visualize the pathways that were activated during the time of infection.

## Additional files

Additional file 1: **Table S1.** Summary of read numbers obtained from soybean plants and *Phytophthora sojae* following inoculation experiments over time on plants treated or not with silicon (Si). Total read numbers and read numbers aligned onto soybean and *P. sojae* genomes are given in millions ± SE. (DOCX 14 kb)

Additional file 2: **Table S2.** Normalized expression of differentially expressed genes in soybean roots infected with *Phytophthora sojae.* In total, 3294 genes were differentially expressed as a result of *P. sojae* infection at day 4 after inoculation (P^+^Si^−^). The normalized mean (*n* = 5) of the differentially expressed transcripts (Fold-change ≥4, FDR *p*-value ≤0.01) are presented in soybean roots inoculated (P^+^) or not (P^−^) with *P. sojae* and treated (Si^+^) or not (Si^−^) with silicon. (XLSB 215 kb)

Additional file 3: **Figure S1.** Expression profile of signaling-related genes***.*** Gene expression shows a higher expression of 46 receptor kinase family genes (a), 24 RLK genes (b), 5 MAPKs genes (c), and 33 CDPKs genes (d) in *Phytophthora sojae*-inoculated soybean plants at 4 dpi under Si^−^ compared to Si^+^ treatment. Graph shows the average relative (%) expression at each timepoint based on the highest level of expression for each gene as a measure to showcase the trend in expression dynamics. Bars represent standard error from the mean (*n* = 5). (TIF 371 kb)

Additional file 4: **Table S3.** Expression data of signaling-related genes. Normalized mean (*n* = 5), fold-change and FDR *p*-value of 46 receptor kinase family genes (sheet 1), 24 RLK genes (sheet 2), 5 MAPKs genes (sheet 3), and 33 CDPKs genes (sheet 4) in soybean plants treated (Si+) or not (Si-) with silicon (Si) at 0, 4, 7, and 14 dpi with *Phytophthora sojae. (XLSX 65 kb)*

Additional file 5: **Table S4.** Expression data of *NB-LRRs* genes. Normalized mean (*n* = 5), fold-change and FDR *p*-value of 45 receptor (*NB-LRR*) genes in soybean plants treated (Si+) or not (Si-) with silicon (Si) at 0, 4, 7, and 14 dpi with *Phytophthora sojae*. (XLSX 22 kb)

Additional file 6: **Table S5.** Expression data of *PR* genes. Normalized mean (*n* = 5), fold-change and FDR *p*-value of 11 pathogenesis-related (*PR*) genes in soybean plants treated (Si+) or not (Si-) with silicon (Si) at 0, 4, 7, and 14 dpi with *Phytophthora sojae*. (XLSX 12 kb)

Additional file 7: **Figure S2.** Expression profile of WRKY transcription factor genes. Gene expression shows a higher expression of 20 WRKY genes in *Phytophthora sojae*-inoculated soybean plants at 4 dpi under Si^−^ compared to Si^+^ treatment. Graph shows the average relative (%) expression at each timepoint based on the highest level of expression for each gene as a measure to showcase the trend in expression dynamics. Bars represent standard error from the mean (*n* = 5). (TIF 86 kb)

Additional file 8: **Table S6.** Expression data of *WRKY* transcription factor genes. Normalized mean (*n* = 5), fold-change and FDR *p*-value of 20 *WRKY* genes in soybean plants treated (Si+) or not (Si-) with silicon (Si) at 0, 4, 7, and 14 dpi with *Phytophthora sojae*. (XLSX 16 kb)

Additional file 9: **Figure S3.** Expression profile of a) protease inhibitors and b) polyphenol oxidase. Gene expression shows a higher expression of 13 protease inhibitor genes (a) and five polyphenol oxidase genes (b) in *Phytophthora sojae*-inoculated soybean plants at 4 dpi under Si^−^ compared to Si^+^ treatment. Graph shows the average relative (%) expression at each timepoint based on the highest level of expression for each gene as a measure to showcase the trend in expression dynamics. Bars represent standard error from the mean (*n* = 5). (TIF 163 kb)

Additional file 10: **Table S7.** Expression data of *a) protease inhibitor* genes *and b) polyphenol oxidase* genes. Normalized mean (*n* = 5), fold-change and FDR *p*-value of 13 *protease inhibitor* genes (sheet 1) and five *polyphenol oxidase* genes (sheet 2) in soybean plants treated (Si+) or not (Si-) with silicon (Si) at 0, 4, 7, and 14 dpi with *Phytophthora sojae*. (XLSX 37 kb)

Additional file 11: **Figure S4.** Expression profile of secondary metabolism-related genes. Gene expression shows a higher expression of 31 genes involved in flavonoid metabolism (a), nine genes involved in isoflavone metabolism (b) and 15 genes involved in isoprenoid metabolism (c) in *Phytophthora sojae*-inoculated soybean plants at 4 dpi under Si^−-^ compared to Si^+^ treatment. Graph shows the average relative (%) expression at each timepoint based on the highest level of expression for each gene as a measure to showcase the trend in expression dynamics. Bars represent standard error from the mean (*n* = 5). (TIF 261 kb)

Additional file 12: **Table S8.** Expression data of secondary metabolism-related genes. Normalized mean (*n* = 5), fold-change and FDR *p*-value of 31 genes involved in flavonoid metabolism (sheet 1), nine genes involved in isoflavone metabolism (sheet 2) and 15 genes involved in isoprenoid metabolism (sheet 3) in soybean plants treated (Si+) or not (Si-) with silicon (Si) at 0, 4, 7, and 14 dpi with *Phytophthora sojae*. (XLSX 48 kb)

Additional file 13: **Table S9.** Expression data of hormone-related genes. Normalized mean (*n* = 5), fold-change and FDR *p*-value of five genes involved in SA metabolism (sheet 1), and13 genes involved in JA metabolism (sheet 2) in soybean plants treated (Si+) or not (Si-) with silicon (Si) at 0, 4, 7, and 14 dpi with *Phytophthora sojae*. (XLSX 36 kb)

Additional file 14: **Figure S5.** Heat map of differentially expressed genes involved in primary metabolism. Heat map shows gene expression pattern of 580 DEGs involved in primary metabolism in soybean roots inoculated (P^+^) or not (P^−-^) with *P. sojae* and treated (Si^+^) or not (Si^−-^) with silicon showing a notable higher expression of genes in *P. sojae-*infected plants. Each gene corresponds to a colored line indicating the normalized mean (*n* = 5) of the differentially expressed transcripts (Fold-change ≥ 4, FDR *p*-value ≤ 0.01). (TIF 205 kb)

Additional file 15: **Table S10.** Summary of read numbers obtained from the five biological replications of *Phytophthora sojae* in axenic culture. Total read numbers and read numbers aligned onto *P. sojae* genomes are given in millions. (DOCX 13 kb)

Additional file 16: **Table S11.** List of the top 100 upregulated *Phytophthora sojae* genes during the compatible interaction with soybean plants at 4, 7 and 14 dpi. (DOCX 86 kb)

## Abbreviations

*Avr*: Avirulence
CDPKs: Ca^2+^-Dependent Protein Kinases
CRN: Crinkler
CSEPs: Candidate Secreted Effector Proteins
DEGs: Number of Differentially Expressed Genes
dpi: Days Post Inoculation
ETI: Effector-Triggered Immunity
FDR: False Discovery Rate
GO: Gene Ontology
JA: Jasmonic Acid
MAPKs: Mitogen-Activated Protein Kinases
NB-LRR: Nucleotide-Binding Leucine-Rich Repeat
*P. sojae*: *Phytophthora sojae*
PAMPs: Pathogen-Associated Molecular Patterns
PRRs: Pattern Recognition Receptors
PRs: Pathogenesis-Related proteins
PTI: PAMPs -Triggered Immunity
RLK: Receptor-Like Kinases
*Rps*: Resistance genes to *P. sojae*
SA: Salicylic Acid
Si: Silicon

## References

[1] Arsenault-Labrecque G, Menzies JG, Bélanger RR (2012). Effect of silicon absorption on soybean resistance to Phakopsora pachyrhizi in different cultivars. Plant Dis.

[2] Bauer P, Elbaum R, Weiss IM (2011). Calcium silicon minerelization in land plants: transport, structure and function. Plant Sci.

[3] Bélanger RR, Benhamou N, Menzies JG (2003). Cytological evidence of an active role of silicon in wheat resistance to powdery mildew (Blumeria graminis f. sp. tritici). Phytopathology.

[4] Bozkurt TO, Schornack S, Banfield MJ, Kamoun S (2012). Oomycetes, effectors, and the all that jazz. Curr Opin in Plant Biol.

[5] Chain F, Côté-Beaulieu C, Belzile F, Menzies JG, Bélanger RR (2009). A Comprehensive transcriptomic analysis of the effect of silicon on wheat plants under control and pathogen stress conditions. Mol Plant-Microbe Interact.

[6] Chérif M, Asselin A, Bélanger RR (1994). Defense responses induced by soluble silicon in cucumber roots infected by Pythium spp. Phytopathology.

[7] Chérif M, Benhamou N, Menzies JG, Bélanger RR (1992). Silicon induced resistance in cucumber plants against Pythium ultimum. Physiol Mol Plant Pathol.

[8] Chérif M, Menzies JG, Benhamou N, Bélanger RR (1992). Studies of silicon distribution in wounded and Pythium ultimum infected cucumber plants. Physiol Mol Plant Pathol.

[9] Deshmukh RK, Vivancos J, Guérin V, Sonah H, Labbé C, Belzile F, Bélanger RR (2013). Identification and functional characterization of silicon transporters in soybean using comparative genomics of major intrinsic proteins in Arabidopsis and rice. Plant Mol Biol.

[10] Dodds PN, Rathjen JP (2010). Plant immunity: towards an integrated view of plant-pathogen interactions. Nat Rev Genet.

[11] Dong S, Yu D, Cui L, Quitob D, Tedman J, Kale SD, Tyler B, Wang Y, Gijzen M, Yang CH (2011). Sequence variants of the Phytophthora sojae RxLR effector Avr3a/5 are differentially recognized by *Rps3a* and *Rps5* in soybean. PLoS One.

[12] Dou D, Kale SD, Wang X, Jiang RHY, Bruce NA, Arredondo FD, Zhang X, Tyler BM (2008). RxLR-mediated entry of Phytophthora sojae effector Avr1b into soybean cells does not require pathogen-encoded machinery. Plant Cell.

[13] Du Z, Zhou X, Ling Y, Zhang Z, Su Z (2010). AgriGO: a GO analysis toolkit for the agricultural community. Nucleic Acids Res.

[14] Fang Z, Cui X (2011). Design and validation issues in RNA-seq experiments. Brif Bioinform.

[15] Fauteux F, Chain F, Belzile F, Menzies JG, Bélanger RR (2006). The protective role of silicon in the Arabidopsis–powdery mildew pathosystem. PNAS.

[16] Fauteux F, Rémus-Borel W, Menzies JG, Bélanger RR (2005). Silicon Plant disease resistance against pathogenic fungi. FEMS Microbiol Lett.

[17] Fawe A, Abou-Zaid M, Menzies JG, Bélanger RR (1998). Silicon-mediated accumulation of flavonoid phytoalexins in cucumber. Phytopathology.

[18] Fliegmann J, Mithofer A, Wanner G, Ebel J (2004). An ancient enzyme domain hidden in the putative beta-glucan elicitor receptor of soybean may play an active part in the perception of pathogen-associated molecular patterns during broad host resistance. J Biol Chem.

[19] Gao H, Narayanan NN, Ellison L, Hattacharyya MK (2005). Two classes of highly similar coiled coil-nucleotide binding-leucine rich repeat genes isolated from the Rps1-k locus encode Phytophthora resistance in soybean. Mol Plant Microbe Interact.

[20] Ghanmi D, McNally DJ, Benhamou N, Menzies JG, Bélanger RR (2004). Powdery mildew of Arabidopsis thaliana: a pathosystem for exploring the role of silicon in plant–microbe interactions. Physiol Mol Plant Pathol.

[21] Glazebrook J (2005). Contrasting mechanisms of defense against biotrophic and necrotrophic pathogens. Annu Rev Phytopathol.

[22] Guérin V, Lebreton A, Cogliati EE, Hartley SE, Belzile F, Menzies JG, Bélanger RR (2014). A zoospore inoculation method with Phytophthora sojae to assess the prophylactic role of silicon on soybean cultivars. Plant Dis.

[23] Guével MH, Menzies JG, Bélanger RR (2007). Effect of root and foliar applications of soluble silicon on powdery mildew control and growth of wheat plants. Eur J Plant Pathol.

[24] Gunzter F, Keller C, Meunier JD (2012). Benefits of plant silicon for crops: a review. Agron Sustain Dev.

[25] Hacquard S, Kracher B, Maekawa T, Vernaldi S, Schulze-Lefert P (2013). Mosaic genome structure of the barley powdery mildew pathogen and conservation of transcriptional programs in divergent hosts. Proc Natl Acad Sci.

[26] Jones JDG, Dangl JL (2006). The plant immune system. Nature.

[27] Kaufman MJ, Gerdemann JW (1985). Root stem rot of soybean caused by Phytophthora sojae n. sp. Phytopathology.

[28] Kirankumar SM, Choong R (2004). Non- host resistance: how much do we know?. Trends Plants Sci.

[29] Lanubile A, Muppirala UK, Severin AJ, Marocco A, Munkvold G (2015). Transcriptome profiling of soybean (Glycine max) roots challenged with pathogenic and non-pathogenic isolates of Fusarium oxysporum. BMC Genomics.

[30] Lebreton A, Labbé C, De Ronne M, Xue A, Marchand G, Bélanger RR (2018). Development of a simple hydroponic assay to study vertical and horizontal resistance of soybean and pathotypes of Phytophthora sojae. Plant Dis.

[31] Lin F, Zhao M, Douglas DB (2014). Molecular response to the pathogen Phytophthora sojae among ten soybean near isogenic lines revealed by comparative transcriptomics. BMC Genomics.

[32] Liu P, Wei W, Ouyang S, Zhang JS, Chen SY, Zhang WK (2009). Analysis of expressed receptor-like kinases (RLKs) in soybean. J Genet Genomics.

[33] Ma JF, Nishimura K, Takahashi E (1989). Effect of silicon on the growth of rice plant at different growth stage. Soil Sci Plant Nutr.

[34] Ma Z, Lin Z, Tianqiaoc S, Yang W, Qi Z, Yeqiang X, Min Q, Yachun L, Haiyang L, Liang K, Yufeng F, Wenwu Y, Yan W, Suomeng D, Xiaobo Z, Brett T, Yuanchao W (2017). A Paralogous decoy protects Phytophthora sojae apoplastic effector PsXEG1 from a host inhibitor. Science.

[35] Meena VD, Dotaniya ML, Coumar V, Rajendiran S, Ajay S, Kundu S, Rao AS (2014). A case for silicon fertilization to improve crop yields in tropical soils. Proc Natl Acad Sci India B.

[36] Moy P, Qutob D, Chapman BP, Atkinson I, Gijzen M (2004). Patterns of gene expression upon infection of soybean plants by Phytophthora sojae. Mol Plant Micobe Interact.

[37] Petre B, Kamoun S (2014). How do filamentous pathogens deliver effector proteins into plant cells?. PLoS Biol.

[38] Radwan O, Liu Y, Clough J (2011). Transcriptional analysis of soybean root response to Fusarium virguliforme*,* the casual agent of sudden death syndrome. Mol Plant-Microbe Interact.

[39] Robinson MD, Smyth GK (2008). Small-sample estimation of negative binomial dispersion, with applications to SAGE data. Biostatistics.

[40] Rodrigues FÁ, McNally DJ, Datnoff LE, Jones JB, Labbé C, Benhamou N, Bélanger RR (2004). Silicon enhances the accumulation of diterpenoid phytoalexins in rice: a potential mechanism for blast resistance. Phytopathology.

[41] Rojas CM, Senthil-Kumar M, Tzin V, Mysore KS. Regulation of primary plant metabolism during plant-pathogen interactions and its contribution to plant defense. Front Plant Sci. 2014;5:17. 10.3389/fpls.2014.00017 PMC3919437 24575102

[42] Sonah H, Zhang X, Deshmukh RK, Borhan H, Fernando D, Bélanger RR (2016). Comparative transcriptomic analysis of virulence factors in Leptosphaeria maculans during compatible and incompatible interactions with canola. Front Plant Sci.

[43] Thimm O, Blaesing OE, Gibonv Y, Nagel A, Meyer S, Krueger P, Selbig J, Mueller LA, Rhee SY, Stitt M (2004). MAPMAN: a user-driven tool to display genomics datasets onto diagrams of metabolic pathways and other biological processes. Plant J.

[44] Thordal-Christensen H (2003). Fresh insights into processes of non-host resistance. Curr Opin Plant Biol.

[45] Tyler BM (2007). Phytophthora sojae: root rot pathogen of soybean and model oomycete. Mol Plant Pathol.

[46] Vivancos J, Labbé C, Menzies JG, Bélanger RR (2015). Silicon-mediated resistance of Arabidopsis against powdery mildew involves mechanisms other than the salicylic acid (SA) -dependent defence pathway. Mol Plant Pathol.

[47] Wagner F (1940). Die Bedeutung der Kieselsäure für das Wachstum einiger Kulturpflanzen, ihren Nährstoffhaushalt und ihre Anfälligkeit gegen echte Mehltaupilze. Phytopathol Z.

[48] Wang HL, Li CH, Liang YC, Datnoff LE, Synder GH, Kornodorfer GH (2001). Agricultural utilization of silicon in China. Silicon in agriculture.

[49] Wang Q, Han C, Ferreira AO, Yu W, Tripathy S, Kale SD, Gu B, Sheng Y, Sui Y, Wang X, Zhang Z, Cheng B, Dong S, Shan W, Zheng X, Dou D, Tyler B, Wang Y (2011). Transcriptional programming and functional interactions within the Phytophthora sojae RxLR effector repertoire. Plant Cell.

[50] Wang Y, Wang Y (2018). Trick or treat: microbial pathogens evolved apoplastic effectors modulating plant susceptibility to infection. Mol Plant Microbe Interact.

[51] Watanabe S, Shimoi E, Ohkama N, Hayashi H, Yoneyama T, Yazaki J, Fujii F, Shinbo K, Yamamoto K, Sakata K, Sasaki T, Kishimoto N, Kikuchi S, Fujiwara T (2004). Identification of several rice genes regulated by Si nutrition. Soil Sc Plant Nutr.

[52] Xin XF, Nomura K, Aung K, Velásquez AC, Yao J, Boutrot F, Chang JH, Zipfel C, Sheng Yang H (2016). Bacteria establish an aqueous living space in plants crucial for virulence. Nature.

[53] Ye M, Song Y, Long J, Wang R, Baerson SR, Pan Z, Zhu-Salzman K, Xie J, Cai K, Luo S, Zeng R (2013). Priming of jasmonate-mediated antiherbivore defense responses in rice by silicon. P Natl Acad Sci.

[54] Yoshi H (1941). Studies on the nature of rice blast resistance. Kyusu Imp Univ Sci Fakultato Terkultura Bull.

[55] Zhang C, Wang L, Zhang W, Zhang F (2013). Do lignification and silicification of the cell wall precede silicon deposition in the silica cell of the rice (Oryza sativa L.) leaf epidermis?. Plant Soil.

