# Supplementary material for: Silicon protects soybean plants against Phytophthora sojae by interfering with effector-receptor expression
Source: BMC Plant Biol. 2018 May 30;18:97. doi: 10.1186/s12870-018-1312-7 (PMC5977513; doi:10.1186/s12870-018-1312-7)
Supplement: Supplementary file 1 — Table S1. Summary of read numbers obtained from soybean plants and Phytophthora sojae following inoculation experiments over time on plants treated or not with silicon (Si). Total read numbers and read numbers aligned onto soybean and P. sojae genomes are given in millions ± SE. (DOCX 14 kb) [file 12870_2018_1312_MOESM1_ESM.docx]

Table S1. Summary of read numbers obtained from soybean plants and *Phytopthora sojae* following inoculation experiments over time on plants treated or not with silicon (Si). Total read numbers and read numbers aligned onto soybean and *P. sojae* genomes are given in millions ± SE.

|  | **Control** | | **Soybean –*P.sojae* interaction** | | | | | |
| --- | --- | --- | --- | --- | --- | --- | --- | --- |
|  |  |  | **Si-** | | | **Si+** | | |
|  | **Si-** | **Si+** | **4DPI** | **7DPI** | **14DPI** | **4DPI** | **7DPI** | **14DPI** |
| **Total Reads** | 27± 4 | 22 ± 1 | 18 ± 3 | 17.5 ± 5 | 16 ± 1.5 | 16.5 ± 3 | 16 ± 6 | 16 ± 1.5 |
| **Soybean Mapped Reads** | 22± 3 | 20± 0.8 | 11± 2.5 | 12.5± 0.7 | 12.2±1 | 12.6 ± 0.6 | 12 ± 0.6 | 12± 1 |
| ***P. sojae* Mapped Reads** |  |  | 3 ± 0.6 | 3.4 ± 0.7 | 3.5 ± 0.8 | 2 ± 0.05 | 2.5 ± 0.6 | 2.8 ± 1 |
| **Un mapped** | 4.6 | 2.3 | 3.3 | 1.2 | 0.01 | 1.8 | 1.2 | 1.2 |
