# Supplementary figures and images for: Silicon protects soybean plants against Phytophthora sojae by interfering with effector-receptor expression
Source: BMC Plant Biol. 2018 May 30;18:97. doi: 10.1186/s12870-018-1312-7 (PMC5977513; doi:10.1186/s12870-018-1312-7)

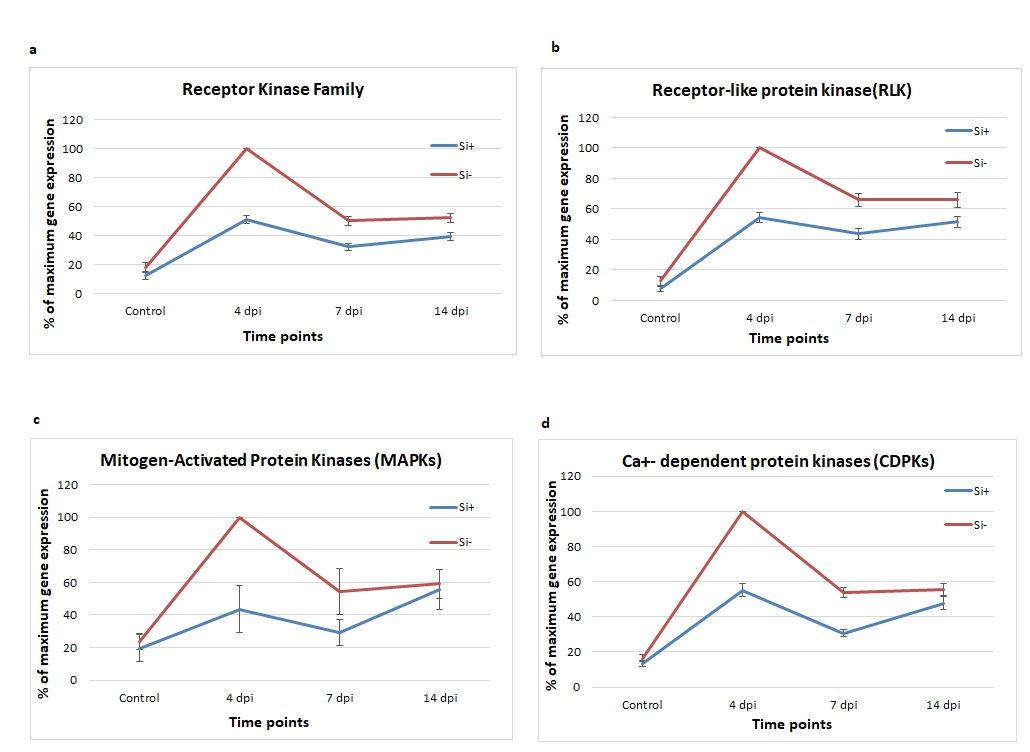

Supplement: Supplementary file 3 — Figure S1. Expression profile of signaling-related genes. Gene expression shows a higher expression of 46 receptor kinase family genes (a), 24 RLK genes (b), 5 MAPKs genes (c), and 33 CDPKs genes (d) in Phytophthora sojae-inoculated soybean plants at 4 dpi under Si− compared to Si+ treatment. Graph shows the average relative (%) expression at each timepoint based on the highest level of expression for each gene as a measure to showcase the trend in expression dynamics. Bars represent standard error from the mean (n = 5). (TIF 371 kb) [file 12870_2018_1312_MOESM3_ESM.tif]

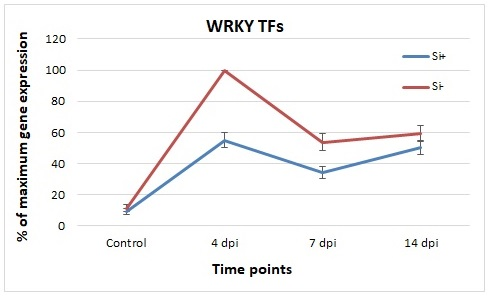

Supplement: Supplementary file 7 — Figure S2. Expression profile of WRKY transcription factor genes. Gene expression shows a higher expression of 20 WRKY genes in Phytophthora sojae-inoculated soybean plants at 4 dpi under Si− compared to Si+ treatment. Graph shows the average relative (%) expression at each timepoint based on the highest level of expression for each gene as a measure to showcase the trend in expression dynamics. Bars represent standard error from the mean (n = 5). (TIF 86 kb) [file 12870_2018_1312_MOESM7_ESM.tif]

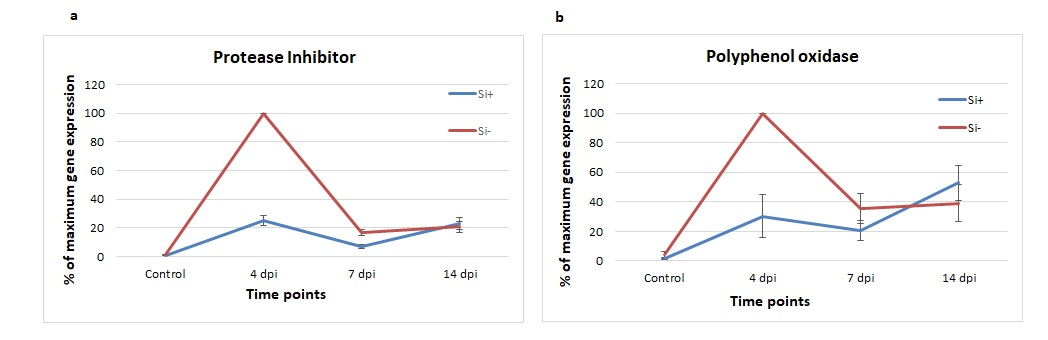

Supplement: Supplementary file 9 — Figure S3. Expression profile of a) protease inhibitors and b) polyphenol oxidase. Gene expression shows a higher expression of 13 protease inhibitor genes (a) and five polyphenol oxidase genes (b) in Phytophthora sojae-inoculated soybean plants at 4 dpi under Si− compared to Si+ treatment. Graph shows the average relative (%) expression at each timepoint based on the highest level of expression for each gene as a measure to showcase the trend in expression dynamics. Bars represent standard error from the mean (n = 5). (TIF 163 kb) [file 12870_2018_1312_MOESM9_ESM.tif]

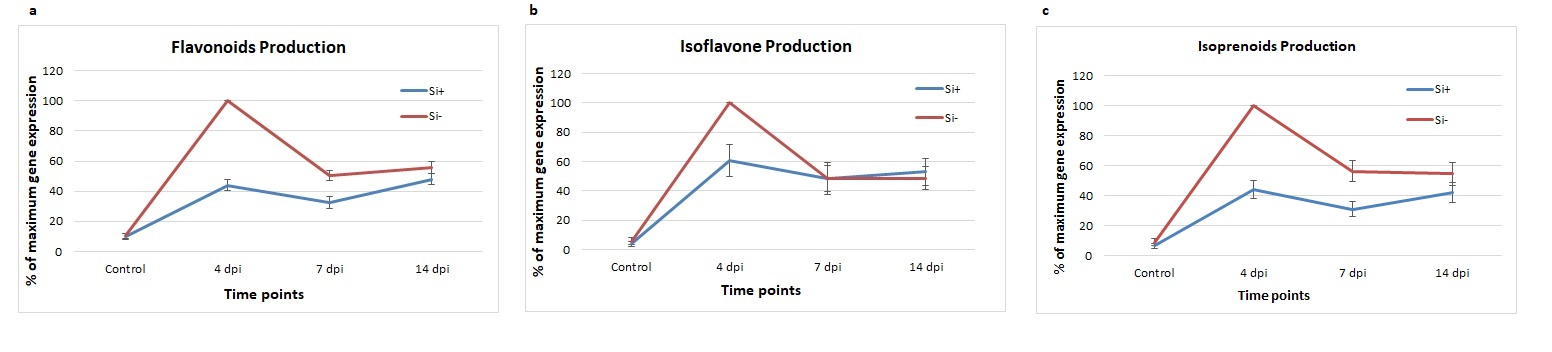

Supplement: Supplementary file 11 — Figure S4. Expression profile of secondary metabolism-related genes. Gene expression shows a higher expression of 31 genes involved in flavonoid metabolism (a), nine genes involved in isoflavone metabolism (b) and 15 genes involved in isoprenoid metabolism (c) in Phytophthora sojae-inoculated soybean plants at 4 dpi under Si−- compared to Si+ treatment. Graph shows the average relative (%) expression at each timepoint based on the highest level of expression for each gene as a measure to showcase the trend in expression dynamics. Bars represent standard error from the mean (n = 5). (TIF 261 kb) [file 12870_2018_1312_MOESM11_ESM.tif]

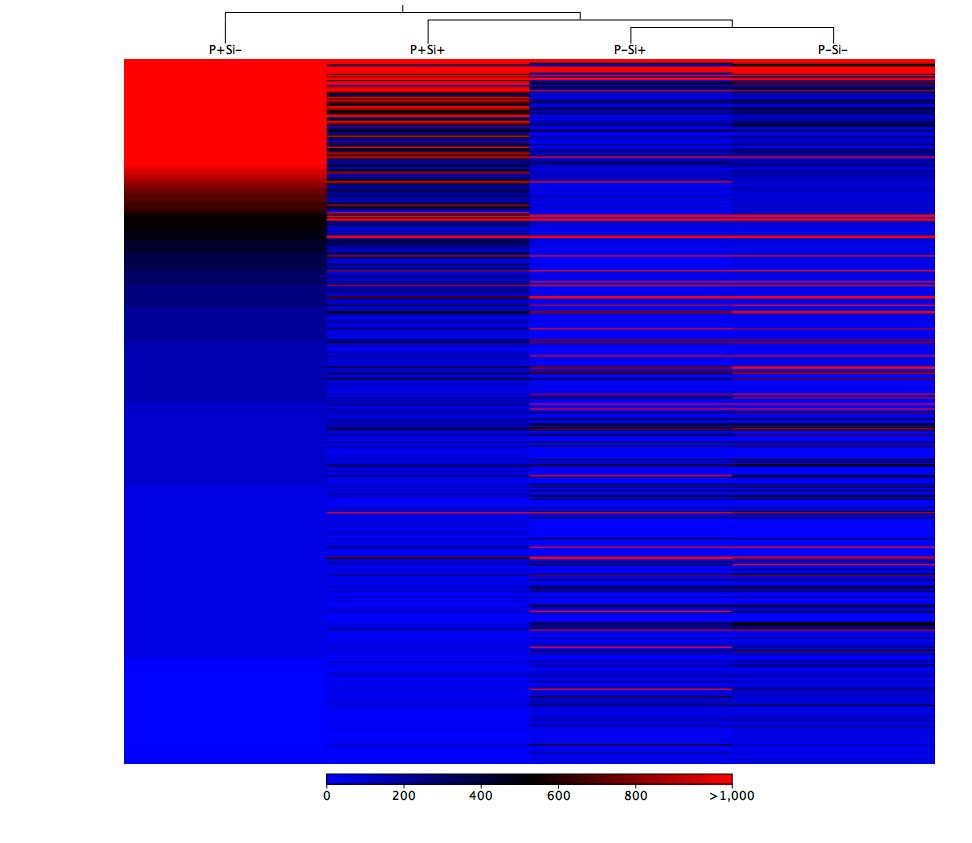

Supplement: Supplementary file 14 — Figure S5. Heat map of differentially expressed genes involved in primary metabolism. Heat map shows gene expression pattern of 580 DEGs involved in primary metabolism in soybean roots inoculated (P+) or not (P−-) with P. sojae and treated (Si+) or not (Si−-) with silicon showing a notable higher expression of genes in P. sojae-infected plants. Each gene corresponds to a colored line indicating the normalized mean (n = 5) of the differentially expressed transcripts (Fold-change ≥ 4, FDR p-value ≤ 0.01). (TIF 205 kb) [file 12870_2018_1312_MOESM14_ESM.tif]
