# Supplementary material for: Silicon protects soybean plants against Phytophthora sojae by interfering with effector-receptor expression
Source: BMC Plant Biol. 2018 May 30;18:97. doi: 10.1186/s12870-018-1312-7 (PMC5977513; doi:10.1186/s12870-018-1312-7)
Supplement: Supplementary file 15 — Table S10. Summary of read numbers obtained from the five biological replications of Phytophthora sojae in axenic culture. Total read numbers and read numbers aligned onto P. sojae genomes are given in millions. (DOCX 13 kb) [file 12870_2018_1312_MOESM15_ESM.docx]

Table S10. Summary of read numbers obtained from the five biological replications of *Phytophthora sojae* in axenic culture. Total read numbers and read numbers aligned onto *P. sojae* genomes are given in millions.

| **Samples** | **Total input reads** | **Total Reads after trimming** | **Mapped Reads on *P. sojae*** | **Percentage** |
| --- | --- | --- | --- | --- |
| **1** | 7,0 | 6,8 | 6,0 | 90 |
| **2** | 6,3 | 6,2 | 5,7 | 92 |
| **3** | 6,2 | 6,1 | 5,7 | 93 |
| **4** | 5,8 | 5,7 | 5,3 | 93 |
| **5** | 6,0 | 5,8 | 5,3 | 90 |
| **Total** | 6,2 ± 0,2 | 6,1 ± 0,2 | 5,6 ± 0,1 | 91,6 ± 0,6 |
