# Supplementary material for: Silicon protects soybean plants against Phytophthora sojae by interfering with effector-receptor expression
Source: BMC Plant Biol. 2018 May 30;18:97. doi: 10.1186/s12870-018-1312-7 (PMC5977513; doi:10.1186/s12870-018-1312-7)
Supplement: Supplementary file 16 — Table S11. List of the top 100 upregulated Phytophthora sojae genes during the compatible interaction with soybean plants at 4, 7 and 14 dpi. (DOCX 86 kb) [file 12870_2018_1312_MOESM16_ESM.docx]

Table S11. List of the top 100 upregulated *Phytopthora sojae* genes during the compatible interaction with soybean plants at 4, 7, and 14 dpi.

| Day | Treatment | Gene ID | Fold Change | FDR p-value correction | GO |
| --- | --- | --- | --- | --- | --- |
| 4 | Si+ | PHYSODRAFT_292717 | 88.885143 | 3.408E-10 | hypothetical protein |
| 4 | Si+ | PHYSODRAFT_291676 | -7.520146 | 8.297E-08 | hypothetical protein |
| 4 | Si+ | PHYSODRAFT_287468 | 15.309291 | 0.0022239 | hypothetical protein |
| 4 | Si+ | PHYSODRAFT_467111 | 4218.0287 | 1.309E-43 | hypothetical protein |
| 4 | Si+ | PHYSODRAFT_548630 | 38.215771 | 8.588E-05 | hypothetical protein |
| 4 | Si+ | PHYSODRAFT_290680 | 60.082027 | 4.514E-05 | hypothetical protein |
| 4 | Si+ | PHYSODRAFT_540605 | 58.339074 | 0.0001464 | hypothetical protein |
| 4 | Si+ | PHYSODRAFT_505018 | 497.48489 | 8.299E-27 | hypothetical protein |
| 4 | Si+ | PHYSODRAFT_311360 | 76.043905 | 3.368E-10 | hypothetical protein |
| 4 | Si+ | PHYSODRAFT_518854 | 1883.1689 | 3.315E-25 | hypothetical protein |
| 4 | Si+ | PHYSODRAFT_558489 | 8363.6512 | 3.831E-27 | hypothetical protein |
| 4 | Si+ | PHYSODRAFT_507388 | 67.543638 | 6.77E-10 | hypothetical protein |
| 4 | Si+ | PHYSODRAFT_349255 | 105.22678 | 1.402E-09 | hypothetical protein |
| 4 | Si+ | PHYSODRAFT_406544 | 4100.8913 | 1.048E-23 | hypothetical protein |
| 4 | Si+ | PHYSODRAFT_545250 | 14.666214 | 0.0055909 | hypothetical protein |
| 4 | Si+ | PHYSODRAFT_297581 | 128152.5 | 5.2E-27 | hypothetical protein |
| 4 | Si+ | PHYSODRAFT_485001 | 163.5942 | 1.2E-11 | hypothetical protein |
| 4 | Si+ | PHYSODRAFT_358957 | 52.055743 | 0.0002422 | hypothetical protein |
| 4 | Si+ | PHYSODRAFT_263134 | 20.232145 | 0.029999 | hypothetical protein |
| 4 | Si+ | PHYSODRAFT_310856 | 49.356267 | 9.427E-05 | hypothetical protein |
| 4 | Si+ | PHYSODRAFT_341906 | 256.52562 | 7.337E-14 | hypothetical protein |
| 4 | Si+ | PHYSODRAFT_535525 | 426.57597 | 7.685E-10 | hypothetical protein |
| 4 | Si+ | PHYSODRAFT_293654 | 61.584757 | 6.754E-07 | hypothetical protein |
| 4 | Si+ | PHYSODRAFT_309735 | 982.3 | 2.06E-21 | hypothetical protein |
| 4 | Si+ | PHYSODRAFT_340057 | -13.77296 | 4.939E-11 | hypothetical protein |
| 4 | Si+ | PHYSODRAFT_502739 | 292.80804 | 7.337E-14 | hypothetical protein |
| 4 | Si+ | PHYSODRAFT_306425 | 167.46124 | 8.152E-06 | hypothetical protein |
| 4 | Si+ | PHYSODRAFT_299117 | 2158.05 | 3.244E-25 | hypothetical protein |
| 4 | Si+ | PHYSODRAFT_555146 | 718.94915 | 4.614E-17 | hypothetical protein |
| 4 | Si+ | PHYSODRAFT_354067 | 25.524346 | 0.0016499 | hypothetical protein |
| 4 | Si+ | PHYSODRAFT_286885 | -16.63318 | 1.502E-11 | hypothetical protein |
| 4 | Si+ | PHYSODRAFT_558669 | 68.617581 | 5.599E-06 | hypothetical protein |
| 4 | Si+ | PHYSODRAFT_346593 | 2155.75 | 5.734E-24 | hypothetical protein |
| 4 | Si+ | PHYSODRAFT_476994 | 5105.3182 | 9.716E-21 | hypothetical protein |
| 4 | Si+ | PHYSODRAFT_301127 | 420.87595 | 8.989E-20 | hypothetical protein |
| 4 | Si+ | PHYSODRAFT_262785 | 134.46363 | 1.943E-11 | hypothetical protein |
| 4 | Si+ | PHYSODRAFT_489480 | 560.25 | 9.362E-20 | hypothetical protein |
| 4 | Si+ | PHYSODRAFT_555774 | 33.27424 | 0.0003592 | hypothetical protein |
| 4 | Si+ | PHYSODRAFT_347587 | -5.594712 | 3.553E-05 | hypothetical protein |
| 4 | Si+ | PHYSODRAFT_360117 | 74.526488 | 7.809E-11 | hypothetical protein |
| 4 | Si+ | PHYSODRAFT_420139 | 70.560712 | 1.381E-07 | hypothetical protein |
| 4 | Si+ | PHYSODRAFT_261944 | 1669.95 | 5.886E-17 | hypothetical protein |
| 4 | Si+ | SOJ6A | -13.42769 | 7.507E-10 | hypothetical protein |
| 4 | Si+ | PHYSODRAFT_355153 | -20.79575 | 1.525E-10 | hypothetical protein |
| 4 | Si+ | PHYSODRAFT_314938 | 7823.1818 | 7.041E-19 | hypothetical protein |
| 4 | Si+ | PHYSODRAFT_286828 | -4.716861 | 9.441E-06 | hypothetical protein |
| 4 | Si+ | PHYSODRAFT_355956 | 59.122172 | 0.0009658 | hypothetical protein |
| 4 | Si+ | PHYSODRAFT_346916 | 84.824668 | 7.199E-07 | hypothetical protein |
| 4 | Si+ | PHYSODRAFT_299370 | 88.554386 | 1.319E-08 | hypothetical protein |
| 4 | Si+ | PHYSODRAFT_493289 | 25.142953 | 0.0020785 | hypothetical protein |
| 4 | Si+ | PHYSODRAFT_492736 | 23948.5 | 1.875E-20 | hypothetical protein |
| 4 | Si+ | PHYSODRAFT_491792 | 147.10515 | 1.177E-05 | hypothetical protein |
| 4 | Si+ | PHYSODRAFT_540813 | 103.75643 | 1.456E-11 | hypothetical protein |
| 4 | Si+ | PHYSODRAFT_338537 | 750.82418 | 4.306E-11 | hypothetical protein |
| 4 | Si+ | PHYSODRAFT_519994 | 254.32386 | 3.704E-11 | hypothetical protein |
| 4 | Si+ | PHYSODRAFT_491698 | 268.518 | 6.78E-14 | hypothetical protein |
| 4 | Si+ | PHYSODRAFT_358973 | -4.203748 | 0.0015561 | hypothetical protein |
| 4 | Si+ | PHYSODRAFT_559981 | 780.01765 | 9.328E-21 | hypothetical protein |
| 4 | Si+ | PHYSODRAFT_502306 | 1089.2 | 7.592E-20 | hypothetical protein |
| 4 | Si+ | PHYSODRAFT_554052 | 217.67979 | 3.209E-14 | hypothetical protein |
| 4 | Si+ | PHYSODRAFT_476490 | 422.09797 | 2.268E-13 | hypothetical protein |
| 4 | Si+ | PHYSODRAFT_286884 | -16.1767 | 1.903E-11 | hypothetical protein |
| 4 | Si+ | PHYSODRAFT_471270 | 1036.975 | 8.828E-16 | hypothetical protein |
| 4 | Si+ | PHYSODRAFT_324166 | 203.61513 | 9.67E-09 | hypothetical protein |
| 4 | Si+ | PHYSODRAFT_477499 | 483.30709 | 8.465E-14 | hypothetical protein |
| 4 | Si+ | PHYSODRAFT_325697 | 15.613898 | 0.0092802 | hypothetical protein |
| 4 | Si+ | PHYSODRAFT_338620 | 817.16667 | 2.017E-21 | hypothetical protein |
| 4 | Si+ | PHYSODRAFT_341316 | 964.525 | 5.485E-16 | hypothetical protein |
| 4 | Si+ | PHYSODRAFT_489196 | 14.91493 | 0.0057979 | hypothetical protein |
| 4 | Si+ | PHYSODRAFT_528260 | 6270 | 2.154E-09 | hypothetical protein |
| 4 | Si+ | PHYSODRAFT_557662 | 37.621324 | 0.0006926 | hypothetical protein |
| 4 | Si+ | PHYSODRAFT_331670 | 6985.875 | 5.831E-15 | hypothetical protein |
| 4 | Si+ | PHYSODRAFT_498306 | 313.32857 | 2.088E-09 | hypothetical protein |
| 4 | Si+ | PHYSODRAFT_483288 | 134.12438 | 4.116E-12 | hypothetical protein |
| 4 | Si+ | PHYSODRAFT_560508 | 22.514351 | 0.0207683 | hypothetical protein |
| 4 | Si+ | PHYSODRAFT_377518 | 121.99768 | 3.18E-08 | hypothetical protein |
| 4 | Si+ | PHYSODRAFT_492973 | 166.2769 | 7.008E-13 | hypothetical protein |
| 4 | Si+ | PHYSODRAFT_293159 | 51394.5 | 3.429E-15 | hypothetical protein |
| 4 | Si+ | PHYSODRAFT_522988 | 170.78788 | 9.202E-13 | hypothetical protein |
| 4 | Si+ | PHYSODRAFT_344784 | 15.383647 | 0.0388721 | hypothetical protein |
| 4 | Si+ | PHYSODRAFT_505563 | 139.70282 | 1.27E-08 | hypothetical protein |
| 4 | Si+ | PHYSODRAFT_330219 | 71.923469 | 1.239E-06 | hypothetical protein |
| 4 | Si+ | PHYSODRAFT_293418 | 66.282401 | 9.877E-06 | hypothetical protein |
| 4 | Si+ | PHYSODRAFT_563553 | 344.13214 | 1.402E-09 | hypothetical protein |
| 4 | Si+ | PHYSODRAFT_256884 | 501.03125 | 2.024E-15 | hypothetical protein |
| 4 | Si+ | PHYSODRAFT_496444 | 821.56034 | 2.685E-13 | hypothetical protein |
| 4 | Si+ | SOJ2C | -13.69707 | 3.964E-11 | hypothetical protein |
| 4 | Si+ | PHYSODRAFT_467666 | 58.327806 | 2.997E-05 | hypothetical protein |
| 4 | Si+ | PHYSODRAFT_308593 | 119.98813 | 1.224E-10 | hypothetical protein |
| 4 | Si+ | PHYSODRAFT_302408 | 97.83944 | 1.993E-07 | hypothetical protein |
| 4 | Si+ | PHYSODRAFT_542669 | 10.13413 | 0.0365248 | hypothetical protein |
| 4 | Si+ | PHYSODRAFT_502202 | 165.2119 | 6.929E-11 | hypothetical protein |
| 4 | Si+ | PHYSODRAFT_374901 | 1706.0769 | 1.099E-17 | hypothetical protein |
| 4 | Si+ | PHYSODRAFT_547717 | 200.31136 | 2.358E-12 | hypothetical protein |
| 4 | Si+ | PHYSODRAFT_555498 | -10.54576 | 2.212E-07 | hypothetical protein |
| 4 | Si+ | PHYSODRAFT_321690 | 405.57143 | 3.331E-18 | hypothetical protein |
| 4 | Si+ | PHYSODRAFT_520913 | 29.490466 | 0.0054012 | hypothetical protein |
| 4 | Si+ | PHYSODRAFT_285976 | -4.987271 | 5.092E-05 | hypothetical protein |
| 4 | Si+ | PHYSODRAFT_287102 | 26.737524 | 0.0028689 | hypothetical protein |
| 4 | Si+ | PHYSODRAFT_333191 | 2023.65 | 5.657E-19 | hypothetical protein |
| 7 | Si+ | PHYSODRAFT_292717 | 84.996601 | 5.317E-13 | hypothetical protein |
| 7 | Si+ | PHYSODRAFT_287468 | 16.521733 | 0.0011846 | hypothetical protein |
| 7 | Si+ | PHYSODRAFT_467111 | 4476.7335 | 4.733E-29 | hypothetical protein |
| 7 | Si+ | PHYSODRAFT_505018 | 740.23189 | 4.733E-29 | hypothetical protein |
| 7 | Si+ | PHYSODRAFT_290680 | 74.306794 | 3.752E-09 | hypothetical protein |
| 7 | Si+ | PHYSODRAFT_507388 | 216.75108 | 2.574E-10 | hypothetical protein |
| 7 | Si+ | PHYSODRAFT_311360 | 58.28306 | 0.000195 | hypothetical protein |
| 7 | Si+ | PHYSODRAFT_535525 | 1424.5709 | 3.036E-11 | hypothetical protein |
| 7 | Si+ | PHYSODRAFT_518854 | 2187.2434 | 1.852E-17 | hypothetical protein |
| 7 | Si+ | PHYSODRAFT_310856 | 118.79121 | 1.057E-05 | hypothetical protein |
| 7 | Si+ | PHYSODRAFT_263134 | 45.215236 | 0.0039361 | hypothetical protein |
| 7 | Si+ | PHYSODRAFT_348819 | 104.54882 | 0.0003695 | hypothetical protein |
| 7 | Si+ | PHYSODRAFT_349255 | 130.96503 | 5.393E-06 | hypothetical protein |
| 7 | Si+ | PHYSODRAFT_262785 | 389.11173 | 2.273E-15 | hypothetical protein |
| 7 | Si+ | PHYSODRAFT_406544 | 4890.7238 | 3.608E-17 | hypothetical protein |
| 7 | Si+ | PHYSODRAFT_341906 | 455.51005 | 1.312E-12 | hypothetical protein |
| 7 | Si+ | PHYSODRAFT_554052 | 940.09309 | 4.948E-17 | hypothetical protein |
| 7 | Si+ | PHYSODRAFT_286885 | -8.750534 | 1.886E-07 | hypothetical protein |
| 7 | Si+ | PHYSODRAFT_297581 | 104350.8 | 1.245E-18 | hypothetical protein |
| 7 | Si+ | PHYSODRAFT_558489 | 4848.78 | 1.698E-21 | hypothetical protein |
| 7 | Si+ | PHYSODRAFT_558669 | 109.04518 | 3.088E-05 | hypothetical protein |
| 7 | Si+ | PHYSODRAFT_485001 | 131.88202 | 8.913E-07 | hypothetical protein |
| 7 | Si+ | PHYSODRAFT_293654 | 82.474423 | 7.583E-06 | hypothetical protein |
| 7 | Si+ | PHYSODRAFT_355153 | -9.729473 | 8.478E-07 | hypothetical protein |
| 7 | Si+ | PHYSODRAFT_306425 | 235.78972 | 1.503E-05 | hypothetical protein |
| 7 | Si+ | SOJ6A | -7.176636 | 1.143E-06 | hypothetical protein |
| 7 | Si+ | PHYSODRAFT_340057 | -10.85884 | 4.059E-07 | hypothetical protein |
| 7 | Si+ | PHYSODRAFT_309735 | 1324.859 | 3.475E-15 | hypothetical protein |
| 7 | Si+ | PHYSODRAFT_476490 | 1119.9489 | 8.929E-13 | hypothetical protein |
| 7 | Si+ | PHYSODRAFT_528260 | 17516.267 | 2.824E-12 | hypothetical protein |
| 7 | Si+ | PHYSODRAFT_420139 | 111.03625 | 1.705E-05 | hypothetical protein |
| 7 | Si+ | PHYSODRAFT_293418 | 218.25399 | 1.085E-13 | hypothetical protein |
| 7 | Si+ | PHYSODRAFT_301127 | 628.30244 | 9.24E-09 | hypothetical protein |
| 7 | Si+ | PHYSODRAFT_360117 | 111.59912 | 1.072E-06 | hypothetical protein |
| 7 | Si+ | PHYSODRAFT_346593 | 2921.5765 | 1.09E-17 | hypothetical protein |
| 7 | Si+ | PHYSODRAFT_555146 | 846.81916 | 6.33E-12 | hypothetical protein |
| 7 | Si+ | PHYSODRAFT_560508 | 59.159747 | 0.0011509 | hypothetical protein |
| 7 | Si+ | PHYSODRAFT_489480 | 753.08478 | 3.752E-09 | hypothetical protein |
| 7 | Si+ | PHYSODRAFT_347587 | -4.136023 | 0.0019086 | hypothetical protein |
| 7 | Si+ | PHYSODRAFT_476994 | 6375.6 | 2.808E-14 | hypothetical protein |
| 7 | Si+ | PHYSODRAFT_305770 | 14501.6 | 1.21E-12 | hypothetical protein |
| 7 | Si+ | PHYSODRAFT_559981 | 1608.5481 | 3.998E-17 | hypothetical protein |
| 7 | Si+ | PHYSODRAFT_491698 | 514.46281 | 7.1E-17 | hypothetical protein |
| 7 | Si+ | PHYSODRAFT_483288 | 294.53199 | 3.13E-12 | hypothetical protein |
| 7 | Si+ | PHYSODRAFT_502739 | 264.81545 | 1.518E-11 | hypothetical protein |
| 7 | Si+ | PHYSODRAFT_314938 | 11335.8 | 2.286E-13 | hypothetical protein |
| 7 | Si+ | PHYSODRAFT_286884 | -9.206832 | 2.037E-05 | hypothetical protein |
| 7 | Si+ | PHYSODRAFT_338537 | 1124.8267 | 1.427E-09 | hypothetical protein |
| 7 | Si+ | PHYSODRAFT_284259 | 38.655309 | 0.0286117 | hypothetical protein |
| 7 | Si+ | PHYSODRAFT_299370 | 110.07365 | 6.321E-06 | hypothetical protein |
| 7 | Si+ | PHYSODRAFT_566314 | 110.8369 | 2.308E-05 | hypothetical protein |
| 7 | Si+ | SOJ2C | -7.558447 | 1.002E-06 | hypothetical protein |
| 7 | Si+ | PHYSODRAFT_330219 | 119.06389 | 2.127E-05 | hypothetical protein |
| 7 | Si+ | PHYSODRAFT_338620 | 1189.8909 | 1.66E-11 | hypothetical protein |
| 7 | Si+ | PHYSODRAFT_475558 | 3018.1385 | 4.814E-12 | hypothetical protein |
| 7 | Si+ | PHYSODRAFT_251156 | 12000.4 | 7.768E-16 | hypothetical protein |
| 7 | Si+ | PHYSODRAFT_346916 | 79.666138 | 0.0001282 | hypothetical protein |
| 7 | Si+ | PHYSODRAFT_518254 | 49.706169 | 0.0008965 | hypothetical protein |
| 7 | Si+ | PHYSODRAFT_502202 | 266.50193 | 5.686E-10 | hypothetical protein |
| 7 | Si+ | SOJ2A | 28.552158 | 0.0125554 | hypothetical protein |
| 7 | Si+ | PHYSODRAFT_522988 | 242.37011 | 3.286E-09 | hypothetical protein |
| 7 | Si+ | PHYSODRAFT_286830 | -4.828319 | 0.0058549 | hypothetical protein |
| 7 | Si+ | PHYSODRAFT_505563 | 187.02196 | 4.05E-09 | hypothetical protein |
| 7 | Si+ | PHYSODRAFT_498306 | 368.38596 | 5.687E-09 | hypothetical protein |
| 7 | Si+ | PHYSODRAFT_492973 | 199.18039 | 1.771E-09 | hypothetical protein |
| 7 | Si+ | PHYSODRAFT_496444 | 1074.9778 | 3.018E-17 | hypothetical protein |
| 7 | Si+ | PHYSODRAFT_307660 | 173.14128 | 1.066E-07 | hypothetical protein |
| 7 | Si+ | PHYSODRAFT_494321 | 236.4958 | 1.569E-08 | hypothetical protein |
| 7 | Si+ | PHYSODRAFT_515065 | 1804.2968 | 2.492E-12 | hypothetical protein |
| 7 | Si+ | PHYSODRAFT_520913 | 39.898923 | 0.0005789 | hypothetical protein |
| 7 | Si+ | PHYSODRAFT_351206 | 208.65509 | 2.648E-06 | hypothetical protein |
| 7 | Si+ | PHYSODRAFT_557662 | 37.043902 | 0.0102563 | hypothetical protein |
| 7 | Si+ | PHYSODRAFT_488117 | 533.88 | 1.938E-09 | hypothetical protein |
| 7 | Si+ | PHYSODRAFT_517198 | 287.92131 | 9.259E-09 | hypothetical protein |
| 7 | Si+ | PHYSODRAFT_518068 | 762.17391 | 1.055E-17 | hypothetical protein |
| 7 | Si+ | PHYSODRAFT_308593 | 140.76324 | 2.637E-06 | hypothetical protein |
| 7 | Si+ | PHYSODRAFT_540813 | 78.229276 | 0.0001888 | hypothetical protein |
| 7 | Si+ | PHYSODRAFT_339348 | 167.70498 | 1.745E-06 | hypothetical protein |
| 7 | Si+ | PHYSODRAFT_347286 | 76.234441 | 0.0001333 | hypothetical protein |
| 7 | Si+ | PHYSODRAFT_492736 | 16382.4 | 4.014E-08 | hypothetical protein |
| 7 | Si+ | PHYSODRAFT_541826 | 27.06843 | 0.0054036 | hypothetical protein |
| 7 | Si+ | PHYSODRAFT_559717 | 33.098421 | 0.0110616 | hypothetical protein |
| 7 | Si+ | PHYSODRAFT_333173 | 1165.44 | 1.162E-07 | hypothetical protein |
| 7 | Si+ | PHYSODRAFT_519749 | 295.58981 | 9.341E-09 | hypothetical protein |
| 7 | Si+ | PHYSODRAFT_484312 | 15376.4 | 5.439E-08 | hypothetical protein |
| 7 | Si+ | PHYSODRAFT_521105 | 157.06335 | 0.0001276 | hypothetical protein |
| 7 | Si+ | PHYSODRAFT_536461 | 2004.4364 | 7.464E-16 | hypothetical protein |
| 7 | Si+ | PHYSODRAFT_458124 | 212.62029 | 3.554E-08 | hypothetical protein |
| 7 | Si+ | PHYSODRAFT_331670 | 6238.2857 | 3.884E-17 | hypothetical protein |
| 7 | Si+ | PHYSODRAFT_319083 | 259.35 | 1.228E-10 | hypothetical protein |
| 7 | Si+ | PHYSODRAFT_358973 | -6.431353 | 5.439E-08 | hypothetical protein |
| 7 | Si+ | PHYSODRAFT_256884 | 459.14348 | 2.1E-13 | hypothetical protein |
| 7 | Si+ | PHYSODRAFT_513062 | 248.08235 | 4.523E-10 | hypothetical protein |
| 7 | Si+ | PHYSODRAFT_557059 | 100.86029 | 6.633E-06 | hypothetical protein |
| 7 | Si+ | PHYSODRAFT_524554 | 34.546952 | 0.0211699 | hypothetical protein |
| 7 | Si+ | PHYSODRAFT_477499 | 348.11 | 2.377E-08 | hypothetical protein |
| 7 | Si+ | PHYSODRAFT_309220 | 29.893813 | 0.0307377 | hypothetical protein |
| 7 | Si+ | PHYSODRAFT_472415 | 838.5551 | 1.58E-11 | hypothetical protein |
| 7 | Si+ | PHYSODRAFT_522084 | 3302.9 | 1.938E-09 | hypothetical protein |
| 7 | Si+ | PHYSODRAFT_308732 | 434.07033 | 6.571E-08 | hypothetical protein |
| 14 | Si+ | PHYSODRAFT_292717 | 90.961535 | 4.427E-25 | hypothetical protein |
| 14 | Si+ | PHYSODRAFT_291676 | -4.48121 | 2.14E-06 | hypothetical protein |
| 14 | Si+ | PHYSODRAFT_287468 | 27.934678 | 1.401E-07 | hypothetical protein |
| 14 | Si+ | PHYSODRAFT_467111 | 3698.7393 | 1.352E-40 | hypothetical protein |
| 14 | Si+ | PHYSODRAFT_290680 | 66.460326 | 1.17E-14 | hypothetical protein |
| 14 | Si+ | PHYSODRAFT_505018 | 578.98965 | 1.707E-17 | hypothetical protein |
| 14 | Si+ | PHYSODRAFT_507388 | 148.1228 | 6.063E-28 | hypothetical protein |
| 14 | Si+ | PHYSODRAFT_476490 | 4100.8962 | 3.965E-29 | hypothetical protein |
| 14 | Si+ | PHYSODRAFT_310856 | 133.69456 | 3.206E-10 | hypothetical protein |
| 14 | Si+ | PHYSODRAFT_311360 | 39.303855 | 0.0001609 | hypothetical protein |
| 14 | Si+ | PHYSODRAFT_360117 | 237.18971 | 3.023E-11 | hypothetical protein |
| 14 | Si+ | PHYSODRAFT_262785 | 352.5096 | 3.137E-22 | hypothetical protein |
| 14 | Si+ | PHYSODRAFT_518854 | 1309.92 | 1.089E-19 | hypothetical protein |
| 14 | Si+ | PHYSODRAFT_559981 | 2967.413 | 4.103E-22 | hypothetical protein |
| 14 | Si+ | PHYSODRAFT_558489 | 5724.7692 | 1.674E-16 | hypothetical protein |
| 14 | Si+ | PHYSODRAFT_348819 | 60.935305 | 1.416E-07 | hypothetical protein |
| 14 | Si+ | PHYSODRAFT_341906 | 327.9637 | 1.08E-13 | hypothetical protein |
| 14 | Si+ | PHYSODRAFT_528260 | 21849.067 | 1.065E-30 | hypothetical protein |
| 14 | Si+ | PHYSODRAFT_535525 | 537.88256 | 1.507E-12 | hypothetical protein |
| 14 | Si+ | PHYSODRAFT_420139 | 124.23319 | 2.832E-14 | hypothetical protein |
| 14 | Si+ | PHYSODRAFT_488117 | 1766.0875 | 1.747E-28 | hypothetical protein |
| 14 | Si+ | PHYSODRAFT_263134 | 18.332046 | 0.0031559 | hypothetical protein |
| 14 | Si+ | PHYSODRAFT_560508 | 69.618088 | 2.374E-05 | hypothetical protein |
| 14 | Si+ | PHYSODRAFT_297581 | 74571.6 | 6.383E-16 | hypothetical protein |
| 14 | Si+ | PHYSODRAFT_554052 | 553.39091 | 6.426E-23 | hypothetical protein |
| 14 | Si+ | PHYSODRAFT_406544 | 2193.9344 | 3.054E-13 | hypothetical protein |
| 14 | Si+ | PHYSODRAFT_349255 | 50.837495 | 2.458E-09 | hypothetical protein |
| 14 | Si+ | PHYSODRAFT_558669 | 75.028806 | 3.307E-10 | hypothetical protein |
| 14 | Si+ | PHYSODRAFT_293654 | 55.336475 | 4.316E-08 | hypothetical protein |
| 14 | Si+ | PHYSODRAFT_483288 | 317.37474 | 1.794E-22 | hypothetical protein |
| 14 | Si+ | PHYSODRAFT_485001 | 82.458129 | 1.938E-05 | hypothetical protein |
| 14 | Si+ | PHYSODRAFT_284752 | 23.279439 | 0.0014967 | hypothetical protein |
| 14 | Si+ | PHYSODRAFT_502739 | 253.42912 | 6.102E-11 | hypothetical protein |
| 14 | Si+ | PHYSODRAFT_305770 | 11767.867 | 7.681E-18 | hypothetical protein |
| 14 | Si+ | PHYSODRAFT_284259 | 43.965378 | 7.773E-05 | hypothetical protein |
| 14 | Si+ | PHYSODRAFT_320359 | 156.66602 | 3.662E-06 | hypothetical protein |
| 14 | Si+ | PHYSODRAFT_347587 | -5.936429 | 1.515E-11 | hypothetical protein |
| 14 | Si+ | PHYSODRAFT_340057 | -19.09009 | 9.599E-27 | hypothetical protein |
| 14 | Si+ | PHYSODRAFT_492973 | 316.4069 | 5.743E-15 | hypothetical protein |
| 14 | Si+ | PHYSODRAFT_505563 | 277.34074 | 2.374E-17 | hypothetical protein |
| 14 | Si+ | PHYSODRAFT_286885 | -22.18164 | 3.01E-13 | hypothetical protein |
| 14 | Si+ | PHYSODRAFT_346916 | 96.72526 | 4.602E-07 | hypothetical protein |
| 14 | Si+ | PHYSODRAFT_360220 | 19.099376 | 0.0312198 | hypothetical protein |
| 14 | Si+ | PHYSODRAFT_555146 | 502.11646 | 1.01E-08 | hypothetical protein |
| 14 | Si+ | PHYSODRAFT_286884 | -12.47564 | 6.787E-08 | hypothetical protein |
| 14 | Si+ | PHYSODRAFT_386027 | 1851.3429 | 2.846E-06 | hypothetical protein |
| 14 | Si+ | PHYSODRAFT_306425 | 99.727763 | 6.836E-06 | hypothetical protein |
| 14 | Si+ | PHYSODRAFT_467666 | 97.318085 | 9.295E-10 | hypothetical protein |
| 14 | Si+ | PHYSODRAFT_491698 | 318.41233 | 9.849E-16 | hypothetical protein |
| 14 | Si+ | PHYSODRAFT_301127 | 307.77167 | 2.328E-18 | hypothetical protein |
| 14 | Si+ | PHYSODRAFT_489480 | 410.58246 | 1.118E-19 | hypothetical protein |
| 14 | Si+ | PHYSODRAFT_473153 | 316.56545 | 1.865E-15 | hypothetical protein |
| 14 | Si+ | PHYSODRAFT_309735 | 577.10847 | 4.923E-18 | hypothetical protein |
| 14 | Si+ | PHYSODRAFT_493289 | 22.94238 | 0.0067641 | hypothetical protein |
| 14 | Si+ | PHYSODRAFT_346593 | 1329.7469 | 1.089E-19 | hypothetical protein |
| 14 | Si+ | PHYSODRAFT_476994 | 3251.88 | 8.812E-20 | hypothetical protein |
| 14 | Si+ | PHYSODRAFT_518254 | 47.329801 | 1.992E-06 | hypothetical protein |
| 14 | Si+ | SOJ2A | 26.92263 | 0.0157332 | hypothetical protein |
| 14 | Si+ | PHYSODRAFT_286828 | -6.042171 | 1.067E-11 | hypothetical protein |
| 14 | Si+ | SOJ6A | -19.85154 | 1.031E-12 | hypothetical protein |
| 14 | Si+ | PHYSODRAFT_496444 | 1143.7154 | 2.463E-22 | hypothetical protein |
| 14 | Si+ | PHYSODRAFT_349825 | 97.775258 | 3.725E-07 | hypothetical protein |
| 14 | Si+ | PHYSODRAFT_541826 | 31.776404 | 0.0003553 | hypothetical protein |
| 14 | Si+ | SOJ2C | -10.98451 | 1.864E-13 | hypothetical protein |
| 14 | Si+ | PHYSODRAFT_299370 | 68.397066 | 2.664E-09 | hypothetical protein |
| 14 | Si+ | PHYSODRAFT_355153 | -31.88251 | 1.853E-24 | hypothetical protein |
| 14 | Si+ | PHYSODRAFT_518068 | 814.76364 | 2.823E-18 | hypothetical protein |
| 14 | Si+ | PHYSODRAFT_335469 | 1151.6348 | 1.102E-16 | hypothetical protein |
| 14 | Si+ | PHYSODRAFT_314938 | 5240.4 | 3.342E-23 | hypothetical protein |
| 14 | Si+ | PHYSODRAFT_254231 | 2034.288 | 2.568E-10 | hypothetical protein |
| 14 | Si+ | PHYSODRAFT_540813 | 80.322275 | 9.779E-11 | hypothetical protein |
| 14 | Si+ | PHYSODRAFT_338620 | 754.78125 | 3.938E-22 | hypothetical protein |
| 14 | Si+ | PHYSODRAFT_333950 | 30.212696 | 2.689E-05 | hypothetical protein |
| 14 | Si+ | PHYSODRAFT_462169 | 1889.664 | 4.498E-20 | hypothetical protein |
| 14 | Si+ | PHYSODRAFT_338943 | 762.58983 | 1.454E-20 | hypothetical protein |
| 14 | Si+ | PHYSODRAFT_293418 | 63.6375 | 3.854E-11 | hypothetical protein |
| 14 | Si+ | PHYSODRAFT_566314 | 54.474593 | 0.0001731 | hypothetical protein |
| 14 | Si+ | PHYSODRAFT_484124 | 5401.35 | 1.487E-10 | hypothetical protein |
| 14 | Si+ | PHYSODRAFT_339348 | 147.9299 | 1.08E-13 | hypothetical protein |
| 14 | Si+ | PHYSODRAFT_466695 | 223.91746 | 1.32E-14 | hypothetical protein |
| 14 | Si+ | PHYSODRAFT_520913 | 29.350993 | 0.0002889 | hypothetical protein |
| 14 | Si+ | PHYSODRAFT_286830 | -8.224248 | 3.424E-12 | hypothetical protein |
| 14 | Si+ | PHYSODRAFT_532724 | 1630.35 | 2.032E-10 | hypothetical protein |
| 14 | Si+ | PHYSODRAFT_565213 | 222.225 | 1.326E-20 | hypothetical protein |
| 14 | Si+ | PHYSODRAFT_555498 | -11.31107 | 5.004E-17 | hypothetical protein |
| 14 | Si+ | PHYSODRAFT_347286 | 58.595077 | 3.079E-10 | hypothetical protein |
| 14 | Si+ | PHYSODRAFT_358973 | -7.179333 | 2.461E-11 | hypothetical protein |
| 14 | Si+ | PHYSODRAFT_319083 | 237.2775 | 1.572E-14 | hypothetical protein |
| 14 | Si+ | PHYSODRAFT_307660 | 117.64937 | 4.976E-08 | hypothetical protein |
| 14 | Si+ | PHYSODRAFT_330063 | 29.664804 | 5.622E-05 | hypothetical protein |
| 14 | Si+ | PHYSODRAFT_492736 | 12167.2 | 9.87E-10 | hypothetical protein |
| 14 | Si+ | PHYSODRAFT_338794 | 588.29508 | 1.412E-10 | hypothetical protein |
| 14 | Si+ | PHYSODRAFT_348294 | 117.28591 | 0.0002197 | hypothetical protein |
| 14 | Si+ | PHYSODRAFT_321690 | 378.26087 | 1.603E-12 | hypothetical protein |
| 14 | Si+ | PHYSODRAFT_343355 | 289.77 | 2.689E-12 | hypothetical protein |
| 14 | Si+ | PHYSODRAFT_380022 | 21.716475 | 0.0300109 | hypothetical protein |
| 14 | Si+ | PHYSODRAFT_297137 | 61.025494 | 1.049E-06 | hypothetical protein |
| 14 | Si+ | PHYSODRAFT_309220 | 24.970419 | 0.000572 | hypothetical protein |
| 14 | Si+ | PHYSODRAFT_559717 | 23.610105 | 0.0070092 | hypothetical protein |
| 14 | Si+ | PHYSODRAFT_285976 | -6.079043 | 3.885E-09 | hypothetical protein |
| 4 | Si- | PHYSODRAFT_292717 | 57.458984 | 1.128E-06 | hypothetical protein |
| 4 | Si- | PHYSODRAFT_467111 | 4174.1817 | 2.795E-31 | hypothetical protein |
| 4 | Si- | PHYSODRAFT_287468 | 11.466334 | 0.047115 | hypothetical protein |
| 4 | Si- | PHYSODRAFT_311360 | 97.936548 | 6.969E-08 | hypothetical protein |
| 4 | Si- | PHYSODRAFT_505018 | 510.07981 | 3.958E-18 | hypothetical protein |
| 4 | Si- | PHYSODRAFT_535525 | 1750.9718 | 2.656E-09 | hypothetical protein |
| 4 | Si- | PHYSODRAFT_518854 | 2686.974 | 3.652E-18 | hypothetical protein |
| 4 | Si- | PHYSODRAFT_558489 | 11676.176 | 3.362E-19 | hypothetical protein |
| 4 | Si- | PHYSODRAFT_548630 | 18.408396 | 0.0133323 | hypothetical protein |
| 4 | Si- | PHYSODRAFT_540605 | 27.537779 | 0.0078716 | hypothetical protein |
| 4 | Si- | PHYSODRAFT_406544 | 6084.6781 | 1.418E-15 | hypothetical protein |
| 4 | Si- | PHYSODRAFT_309735 | 3140.0217 | 7.126E-17 | hypothetical protein |
| 4 | Si- | PHYSODRAFT_349255 | 146.53672 | 1.358E-07 | hypothetical protein |
| 4 | Si- | PHYSODRAFT_290680 | 20.809654 | 0.0156911 | hypothetical protein |
| 4 | Si- | PHYSODRAFT_491792 | 756.43587 | 3.477E-12 | hypothetical protein |
| 4 | Si- | PHYSODRAFT_341906 | 573.30547 | 3.415E-14 | hypothetical protein |
| 4 | Si- | PHYSODRAFT_297581 | 123403.5 | 7.126E-17 | hypothetical protein |
| 4 | Si- | PHYSODRAFT_502739 | 803.04783 | 3.933E-14 | hypothetical protein |
| 4 | Si- | PHYSODRAFT_485001 | 237.67019 | 2.543E-08 | hypothetical protein |
| 4 | Si- | PHYSODRAFT_306425 | 412.75095 | 6.535E-07 | hypothetical protein |
| 4 | Si- | PHYSODRAFT_558669 | 174.5305 | 1.317E-08 | hypothetical protein |
| 4 | Si- | PHYSODRAFT_507388 | 53.032912 | 5.406E-05 | hypothetical protein |
| 4 | Si- | PHYSODRAFT_555146 | 1232.4243 | 4.921E-13 | hypothetical protein |
| 4 | Si- | PHYSODRAFT_476490 | 1273.3077 | 2.319E-12 | hypothetical protein |
| 4 | Si- | PHYSODRAFT_488117 | 1531.9526 | 2.9E-12 | hypothetical protein |
| 4 | Si- | PHYSODRAFT_358957 | 37.912103 | 0.0051753 | hypothetical protein |
| 4 | Si- | PHYSODRAFT_346593 | 2600.9758 | 5.811E-16 | hypothetical protein |
| 4 | Si- | PHYSODRAFT_257507 | 2225.7042 | 5.336E-10 | hypothetical protein |
| 4 | Si- | PHYSODRAFT_348819 | 44.008127 | 0.006351 | hypothetical protein |
| 4 | Si- | PHYSODRAFT_491698 | 556.36882 | 9.189E-19 | hypothetical protein |
| 4 | Si- | PHYSODRAFT_554052 | 474.74242 | 7.498E-14 | hypothetical protein |
| 4 | Si- | PHYSODRAFT_360117 | 95.389722 | 2.865E-08 | hypothetical protein |
| 4 | Si- | PHYSODRAFT_523860 | 287.85529 | 1.713E-10 | hypothetical protein |
| 4 | Si- | PHYSODRAFT_374901 | 4527.5 | 6.634E-12 | hypothetical protein |
| 4 | Si- | PHYSODRAFT_520913 | 83.705021 | 5.175E-05 | hypothetical protein |
| 4 | Si- | PHYSODRAFT_292668 | 403.63542 | 0.0005769 | hypothetical protein |
| 4 | Si- | PHYSODRAFT_338537 | 1192.5928 | 8.145E-09 | hypothetical protein |
| 4 | Si- | PHYSODRAFT_354067 | 20.785758 | 0.0077389 | hypothetical protein |
| 4 | Si- | PHYSODRAFT_559981 | 1049.7158 | 7.356E-19 | hypothetical protein |
| 4 | Si- | PHYSODRAFT_301127 | 368.98302 | 3.983E-11 | hypothetical protein |
| 4 | Si- | PHYSODRAFT_293159 | 95875.5 | 1.408E-10 | hypothetical protein |
| 4 | Si- | PHYSODRAFT_354898 | -4.145251 | 1.298E-05 | hypothetical protein |
| 4 | Si- | PHYSODRAFT_347587 | -6.021684 | 0.0069334 | hypothetical protein |
| 4 | Si- | PHYSODRAFT_521105 | 308.215 | 0.000167 | hypothetical protein |
| 4 | Si- | PHYSODRAFT_331670 | 10151.333 | 4.55E-16 | hypothetical protein |
| 4 | Si- | PHYSODRAFT_360535 | 73.639506 | 0.0023394 | hypothetical protein |
| 4 | Si- | PHYSODRAFT_467477 | 4055.5227 | 3.305E-11 | hypothetical protein |
| 4 | Si- | PHYSODRAFT_293654 | 37.962891 | 0.0032789 | hypothetical protein |
| 4 | Si- | PHYSODRAFT_293391 | 33.959883 | 0.0198441 | hypothetical protein |
| 4 | Si- | PHYSODRAFT_309220 | 60.489881 | 0.0005374 | hypothetical protein |
| 4 | Si- | PHYSODRAFT_310733 | 503.81686 | 1.983E-09 | hypothetical protein |
| 4 | Si- | PHYSODRAFT_537835 | -4.29189 | 0.0081564 | hypothetical protein |
| 4 | Si- | PHYSODRAFT_500351 | 74.177846 | 0.0005316 | hypothetical protein |
| 4 | Si- | PHYSODRAFT_305770 | 6950.875 | 1.095E-15 | hypothetical protein |
| 4 | Si- | PHYSODRAFT_540813 | 120.60751 | 4.865E-09 | hypothetical protein |
| 4 | Si- | PHYSODRAFT_420139 | 56.14714 | 2.925E-05 | hypothetical protein |
| 4 | Si- | PHYSODRAFT_481221 | 57.811636 | 3.853E-05 | hypothetical protein |
| 4 | Si- | PHYSODRAFT_333191 | 3412.3696 | 3.958E-18 | hypothetical protein |
| 4 | Si- | PHYSODRAFT_492736 | 19447.125 | 2.768E-15 | hypothetical protein |
| 4 | Si- | PHYSODRAFT_314175 | 81.863057 | 8.904E-05 | hypothetical protein |
| 4 | Si- | PHYSODRAFT_484312 | 18548.25 | 1.881E-12 | hypothetical protein |
| 4 | Si- | PHYSODRAFT_560508 | 30.479132 | 0.0035123 | hypothetical protein |
| 4 | Si- | PHYSODRAFT_356206 | 46.260568 | 0.0096138 | hypothetical protein |
| 4 | Si- | PHYSODRAFT_377518 | 157.48646 | 3.96E-09 | hypothetical protein |
| 4 | Si- | PHYSODRAFT_333840 | 3855.0833 | 1.509E-09 | hypothetical protein |
| 4 | Si- | PHYSODRAFT_563553 | 465.36986 | 1.179E-09 | hypothetical protein |
| 4 | Si- | PHYSODRAFT_499664 | 853.02532 | 1.177E-10 | hypothetical protein |
| 4 | Si- | PHYSODRAFT_330219 | 95.054533 | 0.0002645 | hypothetical protein |
| 4 | Si- | PHYSODRAFT_296029 | 1111.8 | 2.255E-10 | hypothetical protein |
| 4 | Si- | PHYSODRAFT_486369 | 148.73772 | 1.219E-06 | hypothetical protein |
| 4 | Si- | PHYSODRAFT_321690 | 600.05405 | 1.057E-13 | hypothetical protein |
| 4 | Si- | PHYSODRAFT_345031 | 279.59494 | 1.347E-08 | hypothetical protein |
| 4 | Si- | PHYSODRAFT_498306 | 356.40811 | 3.189E-08 | hypothetical protein |
| 4 | Si- | PHYSODRAFT_328539 | 4887.2308 | 7.035E-13 | hypothetical protein |
| 4 | Si- | PHYSODRAFT_286828 | -6.247032 | 9.875E-06 | hypothetical protein |
| 4 | Si- | PHYSODRAFT_324166 | 196.91129 | 4.377E-07 | hypothetical protein |
| 4 | Si- | PHYSODRAFT_320671 | 485.76 | 1.673E-08 | hypothetical protein |
| 4 | Si- | PHYSODRAFT_380960 | 3457.4118 | 3.597E-07 | hypothetical protein |
| 4 | Si- | PHYSODRAFT_558555 | 213.26568 | 1.983E-09 | hypothetical protein |
| 4 | Si- | PHYSODRAFT_557662 | 38.001316 | 0.0205078 | hypothetical protein |
| 4 | Si- | PHYSODRAFT_338794 | 791.03425 | 4.576E-15 | hypothetical protein |
| 4 | Si- | PHYSODRAFT_467823 | 301.11316 | 5.527E-10 | hypothetical protein |
| 4 | Si- | PHYSODRAFT_286885 | -35.38131 | 1.74E-09 | hypothetical protein |
| 4 | Si- | PHYSODRAFT_547717 | 243.38646 | 2.041E-08 | hypothetical protein |
| 4 | Si- | PHYSODRAFT_346916 | 59.544614 | 4.113E-05 | hypothetical protein |
| 4 | Si- | PHYSODRAFT_483821 | 80.920118 | 2.017E-05 | hypothetical protein |
| 4 | Si- | PHYSODRAFT_492973 | 165.77523 | 2.015E-11 | hypothetical protein |
| 4 | Si- | PHYSODRAFT_262785 | 64.893976 | 1.645E-06 | hypothetical protein |
| 4 | Si- | PHYSODRAFT_293418 | 71.304144 | 1.303E-06 | hypothetical protein |
| 4 | Si- | PHYSODRAFT_310240 | 252.32701 | 8.236E-11 | hypothetical protein |
| 4 | Si- | PHYSODRAFT_518254 | 36.829498 | 0.0005473 | hypothetical protein |
| 4 | Si- | PHYSODRAFT_565213 | 258.55147 | 3.442E-07 | hypothetical protein |
| 4 | Si- | PHYSODRAFT_473812 | 77.755917 | 0.00075 | hypothetical protein |
| 4 | Si- | PHYSODRAFT_314938 | 3950.0769 | 2.628E-14 | hypothetical protein |
| 4 | Si- | Avh1b-81 | 128.24436 | 0.0006678 | Avh1b-81 |
| 4 | Si- | PHYSODRAFT_541826 | 27.26518 | 0.0030735 | hypothetical protein |
| 4 | Si- | PHYSODRAFT_555774 | 15.809186 | 0.029201 | hypothetical protein |
| 4 | Si- | PHYSODRAFT_287102 | 30.713555 | 0.0072605 | hypothetical protein |
| 4 | Si- | PHYSODRAFT_472341 | 197.35625 | 5.836E-09 | hypothetical protein |
| 4 | Si- | PHYSODRAFT_321238 | 2687.1176 | 3.773E-11 | hypothetical protein |
| 7 | Si- | PHYSODRAFT_292717 | 88.73562 | 7.869E-16 | hypothetical protein |
| 7 | Si- | PHYSODRAFT_287468 | 18.975099 | 1.78E-07 | hypothetical protein |
| 7 | Si- | PHYSODRAFT_467111 | 4538.5267 | 2.053E-75 | hypothetical protein |
| 7 | Si- | PHYSODRAFT_505018 | 597.8891 | 1.577E-38 | hypothetical protein |
| 7 | Si- | PHYSODRAFT_311360 | 66.516125 | 1.378E-10 | hypothetical protein |
| 7 | Si- | PHYSODRAFT_476490 | 3804.875 | 5.357E-21 | hypothetical protein |
| 7 | Si- | PHYSODRAFT_290680 | 22.420228 | 0.0056581 | hypothetical protein |
| 7 | Si- | PHYSODRAFT_535525 | 968.49127 | 1.603E-13 | hypothetical protein |
| 7 | Si- | PHYSODRAFT_518854 | 1544.6587 | 4.528E-29 | hypothetical protein |
| 7 | Si- | PHYSODRAFT_507388 | 74.486218 | 2.556E-11 | hypothetical protein |
| 7 | Si- | PHYSODRAFT_558489 | 7539.6293 | 1.306E-29 | hypothetical protein |
| 7 | Si- | PHYSODRAFT_310856 | 82.591556 | 6.531E-07 | hypothetical protein |
| 7 | Si- | PHYSODRAFT_341906 | 446.49494 | 1.01E-29 | hypothetical protein |
| 7 | Si- | PHYSODRAFT_349255 | 89.053968 | 1.427E-12 | hypothetical protein |
| 7 | Si- | PHYSODRAFT_485001 | 164.03721 | 3.091E-16 | hypothetical protein |
| 7 | Si- | PHYSODRAFT_420139 | 163.3791 | 2.12E-19 | hypothetical protein |
| 7 | Si- | PHYSODRAFT_297581 | 109524.6 | 1.103E-25 | hypothetical protein |
| 7 | Si- | PHYSODRAFT_406544 | 3276.5354 | 1.595E-24 | hypothetical protein |
| 7 | Si- | PHYSODRAFT_386027 | 4819.507 | 2.508E-17 | hypothetical protein |
| 7 | Si- | PHYSODRAFT_293418 | 276.83115 | 5.153E-14 | hypothetical protein |
| 7 | Si- | PHYSODRAFT_491698 | 724.74122 | 5.012E-23 | hypothetical protein |
| 7 | Si- | PHYSODRAFT_346916 | 184.56407 | 1.62E-15 | hypothetical protein |
| 7 | Si- | PHYSODRAFT_560508 | 68.327858 | 5.713E-06 | hypothetical protein |
| 7 | Si- | PHYSODRAFT_293654 | 68.521817 | 7.029E-08 | hypothetical protein |
| 7 | Si- | PHYSODRAFT_407691 | 2312.4364 | 2.105E-14 | hypothetical protein |
| 7 | Si- | PHYSODRAFT_263134 | 16.419431 | 0.0310136 | hypothetical protein |
| 7 | Si- | PHYSODRAFT_309735 | 1162.6857 | 6.072E-23 | hypothetical protein |
| 7 | Si- | PHYSODRAFT_488117 | 1394.5615 | 8.291E-25 | hypothetical protein |
| 7 | Si- | PHYSODRAFT_558669 | 82.038873 | 8.326E-10 | hypothetical protein |
| 7 | Si- | PHYSODRAFT_360117 | 103.72627 | 5.14E-20 | hypothetical protein |
| 7 | Si- | PHYSODRAFT_299117 | 2054.3818 | 3.263E-26 | hypothetical protein |
| 7 | Si- | PHYSODRAFT_502739 | 299.75672 | 2.745E-17 | hypothetical protein |
| 7 | Si- | PHYSODRAFT_306425 | 171.84535 | 4.988E-07 | hypothetical protein |
| 7 | Si- | PHYSODRAFT_348819 | 37.633819 | 1.239E-05 | hypothetical protein |
| 7 | Si- | PHYSODRAFT_347587 | -4.917375 | 0 | hypothetical protein |
| 7 | Si- | PHYSODRAFT_559981 | 1371.4843 | 1.638E-34 | hypothetical protein |
| 7 | Si- | PHYSODRAFT_555146 | 627.48772 | 8.632E-16 | hypothetical protein |
| 7 | Si- | PHYSODRAFT_346593 | 2093.5765 | 1.031E-27 | hypothetical protein |
| 7 | Si- | PHYSODRAFT_493289 | 36.41097 | 1.722E-06 | hypothetical protein |
| 7 | Si- | PHYSODRAFT_262785 | 131.77114 | 1.046E-11 | hypothetical protein |
| 7 | Si- | PHYSODRAFT_301127 | 388.10751 | 4.796E-14 | hypothetical protein |
| 7 | Si- | PHYSODRAFT_340057 | -18.34854 | 1.054E-27 | hypothetical protein |
| 7 | Si- | PHYSODRAFT_505563 | 275.06239 | 2.151E-17 | hypothetical protein |
| 7 | Si- | SOJ2A | 39.175579 | 0.0022724 | hypothetical protein |
| 7 | Si- | PHYSODRAFT_305770 | 10098.933 | 3.914E-21 | hypothetical protein |
| 7 | Si- | PHYSODRAFT_559286 | 15.219604 | 0.0420365 | hypothetical protein |
| 7 | Si- | PHYSODRAFT_476994 | 4103.0286 | 9.973E-11 | hypothetical protein |
| 7 | Si- | PHYSODRAFT_286885 | -22.97989 | 7.138E-39 | hypothetical protein |
| 7 | Si- | PHYSODRAFT_554052 | 272.81277 | 5.847E-28 | hypothetical protein |
| 7 | Si- | PHYSODRAFT_473153 | 314.07866 | 5.864E-13 | hypothetical protein |
| 7 | Si- | PHYSODRAFT_368734 | 1080.5091 | 6.04E-22 | hypothetical protein |
| 7 | Si- | PHYSODRAFT_310240 | 359.81538 | 1.405E-14 | hypothetical protein |
| 7 | Si- | PHYSODRAFT_541826 | 38.210143 | 1.438E-05 | hypothetical protein |
| 7 | Si- | PHYSODRAFT_492973 | 218.76656 | 1.08E-17 | hypothetical protein |
| 7 | Si- | PHYSODRAFT_566314 | 78.845854 | 0.0001389 | hypothetical protein |
| 7 | Si- | PHYSODRAFT_333283 | 80.052792 | 0.0001761 | hypothetical protein |
| 7 | Si- | PHYSODRAFT_314938 | 6293.04 | 4.219E-31 | hypothetical protein |
| 7 | Si- | PHYSODRAFT_286828 | -6.100563 | 8.343E-23 | hypothetical protein |
| 7 | Si- | PHYSODRAFT_293391 | 24.791981 | 0.0001501 | hypothetical protein |
| 7 | Si- | PHYSODRAFT_483288 | 151.191 | 2.899E-17 | hypothetical protein |
| 7 | Si- | PHYSODRAFT_309220 | 42.918103 | 4.881E-07 | hypothetical protein |
| 7 | Si- | PHYSODRAFT_338537 | 648.40879 | 5.124E-16 | hypothetical protein |
| 7 | Si- | PHYSODRAFT_284259 | 23.258794 | 0.0182931 | hypothetical protein |
| 7 | Si- | PHYSODRAFT_540813 | 85.637366 | 4.86E-17 | hypothetical protein |
| 7 | Si- | PHYSODRAFT_261944 | 841.74545 | 5.58E-16 | hypothetical protein |
| 7 | Si- | PHYSODRAFT_355956 | 42.566871 | 0.016102 | hypothetical protein |
| 7 | Si- | PHYSODRAFT_513062 | 299.58621 | 9.031E-09 | hypothetical protein |
| 7 | Si- | PHYSODRAFT_498306 | 305.50588 | 2.753E-13 | hypothetical protein |
| 7 | Si- | SOJ2C | -12.02965 | 1.306E-29 | hypothetical protein |
| 7 | Si- | PHYSODRAFT_355153 | -35.17134 | 3.914E-21 | hypothetical protein |
| 7 | Si- | PHYSODRAFT_475558 | 1869.8222 | 4.6E-21 | hypothetical protein |
| 7 | Si- | PHYSODRAFT_475325 | 985.608 | 3.395E-20 | hypothetical protein |
| 7 | Si- | PHYSODRAFT_555498 | -8.94908 | 7.751E-23 | hypothetical protein |
| 7 | Si- | PHYSODRAFT_297137 | 84.354404 | 1.486E-08 | hypothetical protein |
| 7 | Si- | PHYSODRAFT_285976 | -4.310685 | 1.079E-11 | hypothetical protein |
| 7 | Si- | PHYSODRAFT_531325 | 21.459834 | 0.0003276 | hypothetical protein |
| 7 | Si- | PHYSODRAFT_319083 | 269.29535 | 8.317E-21 | hypothetical protein |
| 7 | Si- | PHYSODRAFT_331670 | 6611.4857 | 2.087E-14 | hypothetical protein |
| 7 | Si- | PHYSODRAFT_518254 | 32.969784 | 5.491E-06 | hypothetical protein |
| 7 | Si- | PHYSODRAFT_528260 | 5041.3333 | 6.445E-20 | hypothetical protein |
| 7 | Si- | PHYSODRAFT_286884 | -22.27642 | 7.726E-52 | hypothetical protein |
| 7 | Si- | PHYSODRAFT_467666 | 56.783875 | 3.573E-07 | hypothetical protein |
| 7 | Si- | Avh1b-81 | 114.06332 | 0.002056 | Avh1b-81 |
| 7 | Si- | PHYSODRAFT_520913 | 31.08518 | 0.0013735 | hypothetical protein |
| 7 | Si- | PHYSODRAFT_344784 | 13.307745 | 0.0224782 | hypothetical protein |
| 7 | Si- | PHYSODRAFT_362264 | 314.56119 | 2.936E-11 | hypothetical protein |
| 7 | Si- | PHYSODRAFT_293159 | 42088.8 | 5.819E-24 | hypothetical protein |
| 7 | Si- | PHYSODRAFT_308593 | 112.46452 | 6.088E-14 | hypothetical protein |
| 7 | Si- | PHYSODRAFT_338943 | 638.32381 | 6.445E-20 | hypothetical protein |
| 7 | Si- | PHYSODRAFT_286830 | -8.089529 | 1.497E-26 | hypothetical protein |
| 7 | Si- | PHYSODRAFT_299370 | 47.205755 | 7.633E-10 | hypothetical protein |
| 7 | Si- | PHYSODRAFT_523860 | 96.376471 | 2.014E-07 | hypothetical protein |
| 7 | Si- | PHYSODRAFT_380022 | 24.161553 | 0.0249386 | hypothetical protein |
| 7 | Si- | PHYSODRAFT_321690 | 396.96495 | 1.628E-22 | hypothetical protein |
| 7 | Si- | PHYSODRAFT_339348 | 125.00784 | 4.411E-15 | hypothetical protein |
| 7 | Si- | SOJ6A | -31.97767 | 4.41E-44 | hypothetical protein |
| 7 | Si- | PHYSODRAFT_492736 | 12371.2 | 1.022E-18 | hypothetical protein |
| 7 | Si- | PHYSODRAFT_561641 | 477.70909 | 2.111E-15 | hypothetical protein |
| 7 | Si- | PHYSODRAFT_491792 | 77.317895 | 8.046E-09 | hypothetical protein |
| 7 | Si- | PHYSODRAFT_487070 | 91.893467 | 1.206E-17 | hypothetical protein |
| 14 | Si- | PHYSODRAFT_292717 | 82.402354 | 7.163E-19 | hypothetical protein |
| 14 | Si- | PHYSODRAFT_287468 | 33.124106 | 6.228E-08 | hypothetical protein |
| 14 | Si- | PHYSODRAFT_467111 | 3993.404 | 3.014E-57 | hypothetical protein |
| 14 | Si- | PHYSODRAFT_290680 | 69.314879 | 3.012E-11 | hypothetical protein |
| 14 | Si- | PHYSODRAFT_505018 | 575.35826 | 3.861E-22 | hypothetical protein |
| 14 | Si- | PHYSODRAFT_548630 | 21.220204 | 0.0128398 | hypothetical protein |
| 14 | Si- | PHYSODRAFT_507388 | 134.98629 | 5.866E-22 | hypothetical protein |
| 14 | Si- | PHYSODRAFT_540605 | 33.1656 | 0.0113984 | hypothetical protein |
| 14 | Si- | PHYSODRAFT_535525 | 1312.8818 | 6.612E-13 | hypothetical protein |
| 14 | Si- | PHYSODRAFT_476490 | 3114.3688 | 1.439E-35 | hypothetical protein |
| 14 | Si- | PHYSODRAFT_311360 | 33.320174 | 0.004007 | hypothetical protein |
| 14 | Si- | PHYSODRAFT_310856 | 108.29424 | 1.645E-11 | hypothetical protein |
| 14 | Si- | PHYSODRAFT_341906 | 577.83468 | 4.097E-24 | hypothetical protein |
| 14 | Si- | PHYSODRAFT_518854 | 1604.283 | 9.209E-31 | hypothetical protein |
| 14 | Si- | PHYSODRAFT_349255 | 108.50958 | 1.546E-09 | hypothetical protein |
| 14 | Si- | PHYSODRAFT_262785 | 281.88747 | 2.949E-24 | hypothetical protein |
| 14 | Si- | PHYSODRAFT_263134 | 20.287404 | 0.0082814 | hypothetical protein |
| 14 | Si- | PHYSODRAFT_297581 | 89911 | 7.92E-26 | hypothetical protein |
| 14 | Si- | PHYSODRAFT_528260 | 19332.667 | 3.84E-26 | hypothetical protein |
| 14 | Si- | PHYSODRAFT_406544 | 2657.4375 | 2.435E-23 | hypothetical protein |
| 14 | Si- | PHYSODRAFT_348819 | 48.829398 | 3.225E-07 | hypothetical protein |
| 14 | Si- | PHYSODRAFT_493289 | 56.467686 | 1.238E-07 | hypothetical protein |
| 14 | Si- | PHYSODRAFT_485001 | 109.14756 | 2.461E-07 | hypothetical protein |
| 14 | Si- | PHYSODRAFT_386027 | 3514.9302 | 5.299E-27 | hypothetical protein |
| 14 | Si- | PHYSODRAFT_476994 | 6962.1 | 5.918E-21 | hypothetical protein |
| 14 | Si- | PHYSODRAFT_558489 | 3379.2 | 5.914E-15 | hypothetical protein |
| 14 | Si- | PHYSODRAFT_306425 | 172.70683 | 6.608E-06 | hypothetical protein |
| 14 | Si- | PHYSODRAFT_299117 | 2377.2593 | 1.5E-32 | hypothetical protein |
| 14 | Si- | PHYSODRAFT_347587 | -4.384151 | 5.593E-05 | hypothetical protein |
| 14 | Si- | PHYSODRAFT_488117 | 1225.1373 | 3.833E-24 | hypothetical protein |
| 14 | Si- | PHYSODRAFT_558669 | 72.950935 | 9.669E-08 | hypothetical protein |
| 14 | Si- | PHYSODRAFT_284752 | 26.403907 | 0.0010842 | hypothetical protein |
| 14 | Si- | PHYSODRAFT_491698 | 508.56067 | 1.268E-24 | hypothetical protein |
| 14 | Si- | PHYSODRAFT_560508 | 52.105489 | 0.0008047 | hypothetical protein |
| 14 | Si- | PHYSODRAFT_358957 | 26.624219 | 0.0481752 | hypothetical protein |
| 14 | Si- | PHYSODRAFT_420139 | 81.104197 | 9.203E-14 | hypothetical protein |
| 14 | Si- | PHYSODRAFT_301127 | 454.98374 | 2.081E-25 | hypothetical protein |
| 14 | Si- | PHYSODRAFT_532724 | 4622.9167 | 9.178E-15 | hypothetical protein |
| 14 | Si- | PHYSODRAFT_284259 | 43.642379 | 8.491E-05 | hypothetical protein |
| 14 | Si- | PHYSODRAFT_555498 | -4.281809 | 2.365E-08 | hypothetical protein |
| 14 | Si- | PHYSODRAFT_554052 | 364.84364 | 5.399E-23 | hypothetical protein |
| 14 | Si- | PHYSODRAFT_299370 | 121.77506 | 1.358E-10 | hypothetical protein |
| 14 | Si- | PHYSODRAFT_293654 | 44.277577 | 5.639E-10 | hypothetical protein |
| 14 | Si- | PHYSODRAFT_555146 | 572.97006 | 2.841E-15 | hypothetical protein |
| 14 | Si- | PHYSODRAFT_502739 | 220.21395 | 3.943E-12 | hypothetical protein |
| 14 | Si- | PHYSODRAFT_502306 | 1684.5926 | 6.273E-23 | hypothetical protein |
| 14 | Si- | PHYSODRAFT_309735 | 710.52381 | 5.777E-19 | hypothetical protein |
| 14 | Si- | PHYSODRAFT_467666 | 114.72074 | 1.531E-14 | hypothetical protein |
| 14 | Si- | PHYSODRAFT_349825 | 145.86031 | 1.106E-14 | hypothetical protein |
| 14 | Si- | PHYSODRAFT_338620 | 1271.7612 | 1.178E-32 | hypothetical protein |
| 14 | Si- | PHYSODRAFT_286884 | -11.62102 | 5.226E-13 | hypothetical protein |
| 14 | Si- | PHYSODRAFT_346593 | 1658.44 | 1.102E-26 | hypothetical protein |
| 14 | Si- | PHYSODRAFT_340057 | -21.82677 | 1.456E-19 | hypothetical protein |
| 14 | Si- | PHYSODRAFT_286885 | -25.97132 | 2.081E-25 | hypothetical protein |
| 14 | Si- | PHYSODRAFT_360117 | 55.916042 | 1.245E-08 | hypothetical protein |
| 14 | Si- | PHYSODRAFT_496444 | 1308.7636 | 2.326E-20 | hypothetical protein |
| 14 | Si- | PHYSODRAFT_308593 | 195.54696 | 8.211E-17 | hypothetical protein |
| 14 | Si- | PHYSODRAFT_314938 | 7056.6 | 2.366E-31 | hypothetical protein |
| 14 | Si- | PHYSODRAFT_305770 | 7668.8889 | 2.273E-22 | hypothetical protein |
| 14 | Si- | PHYSODRAFT_559981 | 860.45 | 9.877E-25 | hypothetical protein |
| 14 | Si- | PHYSODRAFT_473153 | 294.06009 | 1.726E-15 | hypothetical protein |
| 14 | Si- | PHYSODRAFT_471270 | 1258.7778 | 2.045E-18 | hypothetical protein |
| 14 | Si- | PHYSODRAFT_522988 | 242.7 | 6.637E-17 | hypothetical protein |
| 14 | Si- | PHYSODRAFT_483288 | 174.20051 | 1.05E-20 | hypothetical protein |
| 14 | Si- | PHYSODRAFT_338943 | 1067.2258 | 5.334E-23 | hypothetical protein |
| 14 | Si- | PHYSODRAFT_555774 | 21.256042 | 0.0123211 | hypothetical protein |
| 14 | Si- | PHYSODRAFT_346916 | 72.110345 | 4.722E-07 | hypothetical protein |
| 14 | Si- | PHYSODRAFT_355153 | -28.4149 | 2.114E-54 | hypothetical protein |
| 14 | Si- | PHYSODRAFT_358973 | -4.385651 | 1.791E-06 | hypothetical protein |
| 14 | Si- | PHYSODRAFT_489480 | 339.77778 | 4.049E-12 | hypothetical protein |
| 14 | Si- | PHYSODRAFT_554330 | 151.5335 | 9.154E-12 | hypothetical protein |
| 14 | Si- | PHYSODRAFT_498345 | 25.654762 | 0.0120375 | hypothetical protein |
| 14 | Si- | PHYSODRAFT_319083 | 354.08333 | 7.467E-18 | hypothetical protein |
| 14 | Si- | PHYSODRAFT_293418 | 84.102273 | 9.806E-14 | hypothetical protein |
| 14 | Si- | PHYSODRAFT_286828 | -6.516347 | 1.625E-17 | hypothetical protein |
| 14 | Si- | SOJ2C | -10.73532 | 1.999E-17 | hypothetical protein |
| 14 | Si- | PHYSODRAFT_540813 | 89.669811 | 2.336E-12 | hypothetical protein |
| 14 | Si- | PHYSODRAFT_505563 | 167.11642 | 6.846E-12 | hypothetical protein |
| 14 | Si- | PHYSODRAFT_498306 | 319.53293 | 6.711E-12 | hypothetical protein |
| 14 | Si- | PHYSODRAFT_321690 | 545.08333 | 4.576E-25 | hypothetical protein |
| 14 | Si- | PHYSODRAFT_339348 | 173.41414 | 3.772E-12 | hypothetical protein |
| 14 | Si- | PHYSODRAFT_518254 | 37.530792 | 6.547E-05 | hypothetical protein |
| 14 | Si- | PHYSODRAFT_338537 | 557.91011 | 1.546E-09 | hypothetical protein |
| 14 | Si- | PHYSODRAFT_520913 | 35.627839 | 3.239E-06 | hypothetical protein |
| 14 | Si- | PHYSODRAFT_541826 | 26.970308 | 0.0033265 | hypothetical protein |
| 14 | Si- | PHYSODRAFT_355956 | 36.964174 | 0.0140839 | hypothetical protein |
| 14 | Si- | PHYSODRAFT_563027 | 226.69652 | 9.915E-12 | hypothetical protein |
| 14 | Si- | PHYSODRAFT_492736 | 15086 | 8.512E-16 | hypothetical protein |
| 14 | Si- | PHYSODRAFT_297137 | 77.330973 | 7.224E-11 | hypothetical protein |
| 14 | Si- | PHYSODRAFT_343403 | 217.59391 | 4.575E-05 | hypothetical protein |
| 14 | Si- | PHYSODRAFT_257855 | -4.456507 | 3.501E-12 | hypothetical protein |
| 14 | Si- | PHYSODRAFT_335469 | 875.83333 | 2.91E-17 | hypothetical protein |
| 14 | Si- | PHYSODRAFT_481221 | 32.051948 | 0.0001249 | hypothetical protein |
| 14 | Si- | PHYSODRAFT_559717 | 29.398739 | 0.0026996 | hypothetical protein |
| 14 | Si- | PHYSODRAFT_531325 | 18.979381 | 0.0151769 | hypothetical protein |
| 14 | Si- | PHYSODRAFT_545238 | 34.452504 | 9.314E-06 | hypothetical protein |
| 14 | Si- | PHYSODRAFT_261944 | 718.51852 | 3.24E-10 | hypothetical protein |
| 14 | Si- | PHYSODRAFT_341316 | 699.40741 | 4.469E-18 | hypothetical protein |
| 14 | Si- | PHYSODRAFT_330063 | 29.853968 | 0.0002573 | hypothetical protein |
| 14 | Si- | PHYSODRAFT_492973 | 124.93023 | 1.018E-09 | hypothetical protein |
